# Ceritinib is a novel triple negative breast cancer therapeutic agent

**DOI:** 10.1186/s12943-022-01601-0

**Published:** 2022-06-29

**Authors:** Shengli Dong, Hassan Yousefi, Isabella Van Savage, Samuel C. Okpechi, Maryl K. Wright, Margarite D. Matossian, Bridgette M. Collins-Burow, Matthew E. Burow, Suresh K. Alahari

**Affiliations:** 1 TYK Medicines, Inc, Zhejiang, People’s Republic of China 313100; 2 Department of Biochemistry and Molecular Biology, LSUHSC School of Medicine, New Orleans, LA 70112 USA; 3 Tulane University School of Medicine, New Orleans, Louisiana, 70118 USA; 4 Department of Medicine, The University of Chicago, Chicago, IL 60637 USA; 5 Stanley S. Scott Cancer Center, LSUHSC School of Medicine, New Orleans, LA 70112 USA

**Keywords:** Androgen receptor, Ceritinib, Enzalutamide, Paclitaxel, RTK/ACK/AR

## Abstract

**Background:**

Triple-negative breast cancers (TNBCs) are clinically aggressive subtypes of breast cancer. TNBC is difficult to treat with targeted agents due to the lack of commonly targeted therapies within this subtype. Androgen receptor (AR) has been detected in 12–55% of TNBCs. AR stimulates breast tumor growth in the absence of estrogen receptor (ER), and it has become an emerging molecular target in TNBC treatment.

**Methods:**

Ceritinib is a small molecule inhibitor of tyrosine kinase and it is used in the therapy of non-small lung cancer patients. Enzalutamide is a small molecule compound targeting the androgen receptor and it is used to treat prostate cancer. Combination therapy of these drugs were investigated using AR positive breast cancer mouse xenograft models. Also, combination treatment of ceritinib and paclitaxel investigated using AR^−^ and AR low mouse xenograft and patient derived xenograft models.

**Results:**

We screened 133 FDA approved drugs that have a therapeutic effect of AR^+^ TNBC cells. From the screen, we identified two drugs, ceritinib and crizotinib. Since ceritinib has a well- defined role in androgen independent AR signaling pathways, we further investigated the effect of ceritinib. Ceritinib treatment inhibited RTK/ACK/AR pathway and other downstream pathways in AR^+^ TNBC cells. The combination of ceritinib and enzalutamide showed a robust inhibitory effect on cell growth of AR^+^ TNBC cells in vitro and in vivo. Interestingly Ceritinib inhibits FAK-YB-1 signaling pathway that leads to paclitaxel resistance in all types of TNBC cells. The combination of paclitaxel and ceritinib showed drastic inhibition of tumor growth compared to a single drug alone.

**Conclusions:**

To improve the response of AR antagonist in AR positive TNBC, we designed a novel combinational strategy comprised of enzalutamide and ceritinib to treat AR^+^ TNBC tumors through the dual blockade of androgen-dependent and androgen-independent AR signaling pathways. Furthermore, we introduced a novel therapeutic combination of ceritinib and paclitaxel for AR negative or AR-low TNBCs and this combination inhibited tumor growth to a great extent. All agents used in our study are FDA-approved, and thus the proposed combination therapy will likely be useful in the clinic.

**Supplementary Information:**

The online version contains supplementary material available at 10.1186/s12943-022-01601-0.

## Introduction

Breast cancer is categorized into luminal A, luminal B, ERBB2/HER2, basal-like and triple-negative (TN) subtypes. Approximately 15–20% of breast cancers are classified as triple negative breast cancers (TNBC). TNBC is clinically characterized as a more aggressive cancer and has a poor prognosis with a five-year overall survival (OS) of 78.5%. It is highly prevalent in African-American [1] and premenopausal women [2] and BRCA1/2 mutation carriers [2]. TNBC patients often develop tumor recurrence, which typically occurs within the first 3 years after diagnosis [2]. Due to low or absent expression level of estrogen receptors (ER), progesterone receptors (PR) and the HER2 protein, endocrine therapies and anti-HER2 antibody treatment such as trastuzumab are ineffective in treating TNBC [3]. As a result, cytotoxic chemotherapy is the backbone of systemic therapy in TNBC. Chemotherapy regimen for TNBC is usually a combination treatment of anthracyclines, alkylators, and taxanes and can be given in a neoadjuvant and/or adjuvant setting. Neoadjuvant chemotherapy is the standard approach to reduce tumor burden and evaluate chemo-efficacy prior to surgical resection in high-risk TNBC, while adjuvant is done after surgery [3]. TNBCs have high inter- and intra-tumor heterogeneity [4] resulting in very different treatment responses to standard chemotherapy. The survival rates for chemo-resistant metastatic TNBC patients have not been improved significantly over the past 30 years, and thus there is a pressing unmet need to develop new innovative strategies to control TNBCs in the clinic.

According to gene expression profiling, TNBC can be classified into two distinct basal-like (BL1 and BL2), a mesenchymal-like and a luminal androgen receptor (LAR) subtype [5]. AR is detected in 12–55% of TNBC tumors [6, 7]. LAR tumors are similar to breast tumors termed molecular apocrine [8], and they constitute approximately 16% of TNBCs. LAR tumors exhibit a 9-fold greater AR protein expression compared to all other subtypes and possess enriched expression of AR downstream targets and coactivators [9]. Interestingly, AR expression has also been detected in non-LAR TNBCs, albeit to low levels [10, 11]. The role of AR is complex in breast cancer: AR possesses anti-tumor activity in estrogen receptor positive (ER^+^) breast cancer. However, AR stimulates tumor cell progression in ER-negative (ER^−^) breast cancers and is becoming an emerging molecular target in TNBC [12, 13].

The TNBC subtype is an umbrella term, consisting of all breast cancers that are not classified as luminal, ER+ or HER2 amplified subtypes [14]. Evaluating further subtypes within TNBC based on molecular characteristics may provide therapeutic insights for unique subtypes of TNBC patients. Using the molecular-based sub-classifications, FDA-approved tumor actionable drugs may be repurposed to treat chemo-resistant subtype-specific TNBC tumors. Bicalutamide and enzalutamide are FDA-approved AR antagonists for prostate cancer treatment [15]. AR^+^ TNBCs respond to these AR antagonists’ treatment in vitro and in vivo [11, 16]. AR targeting therapies with bicalutamide or enzalutamide demonstrated early promise because single-agent AR inhibition exhibited modest efficacy in completed AR^+^ TNBC clinical trials. There are an additional 18 ongoing clinical trials targeting AR inhibition for AR^+^ metastatic or locally advanced AR^+^ TNBC in the United States [14]. Thus, there is an urgent need to improve the efficacy of AR antagonism.

In this study, we found that ceritinib, an FDA-approved drug for lung cancers [17], efficiently inhibited growth of LAR TNBC cells. In addition, we identified Activated CDC42 Kinase 1 (ACK1) as a major ceritinib target in LAR TNBC cells and demonstrated that ceritinib inhibited RTK-ACK1/FAK-AR signaling pathway in LAR TNBC cells. Finally, we developed a novel therapeutic strategy for AR^+^ TNBC tumors and a combination treatment for AR^−^ or AR^low^ TNBCs.

## Results

### Screening for FDA-approved cancer specific drugs with activity against AR^+^ TNBCs

MDA-MB-453 is a representative LAR TNBC cell line according to gene expression profiling [18]. MDA-MB-453 has high AR expression and is responsive to androgen stimulation or anti-androgen treatment [19]. Using MDA-MB-453 as a cell model, we performed a cytotoxicity assay and screened 133 FDA approved cancer specific drugs (Suppl Fig. 1A). Cells were treated with a final concentration of 10 μm of drugs and cell viability was determined by MTT assays (Suppl Fig. 1B). The detailed list of drugs that resulted in < 50% of total cell viability is shown in Suppl Fig. 1C. Interestingly, we found that ceritinib and crizotinib were among the most effective drugs against MDA-MB-453 cells (Suppl Fig. 1C). Ceritinib (LDK378) and crizotinib are second-generation inhibitors of anaplastic lymphoma kinase (ALK). They are FDA-approved drugs for the treatment of ALK^+^ metastatic non–small-cell lung cancer (NSCLC). Ceritinib is designed to target the highly conserved ATP binding site in the active ALK1. Due to the similarity of ATP binding sites in all tyrosine kinases, ceritinib also targets ROS1, IGF-1R, FAK, ACK1/TNK2 and many other tyrosine kinases besides ALK1 [20–22]. The off-target effects of small molecule inhibitors are often an undesired phenomenon in the context of drug development as they are synthesized with the intent to generate selective and potent inhibitors. However, it can be beneficial in the search for novel TNBC treatments since ROS1, ACK1, IGF-1R and FAK are all potential therapeutic targets in TNBCs [23–25]. Since ceritinib targets IGF-1R, ERK1/2, PI3K, FAK and ACK1/TNK2 [20], which are important players in androgen-independent AR signaling pathways, we primarily focused our studies on ceritinib.

### Ceritinib exhibited cytotoxicity in LAR and non-LAR TNBC cells in a dose- and time-dependent manner

We further explored the potential of ceritinib to induce cancer cell death in LAR TNBC cells. Ceritinib treatment killed MDA-MB-453 and another representative LAR cell line, MFM223, in a dose- and time-dependent manner (Suppl Fig. 2A B, C, D). The calculated IC_50_ of ceritinib was 1.19 μM for MDA-MB-453 cells (Suppl Fig. 2B) and 1.24 μM for MFM223 cells (Suppl Fig. 2D). The standard administration for advanced NSCLC is 750 mg/day ceritinib (oral), which results in the mean of the maximal plasma concentration (C_max_) at 1.43 ± 0.37 μM [22]. Therefore, IC_50_ of ceritinib for MDA-MB-453 and MFM 223 is clinically achievable. Ceritinib treatment inhibited colony formation in MDA-MD-231, MDA-MB-453 and SUM185PE (Suppl Fig. 3A, B). Because TNBC is a highly heterogeneous cancer, we additionally examined the efficacy of ceritinib on four different human TNBC cell lines. Ceritinib efficiently killed another LAR cell line SUM185PE and three non-LAR TNBC cells MDA-MB-231, MDA-MB 157 and MDA-MB 468 in a dose- and time-dependent manner (Suppl Fig. 4 A, B, C, and D).

### ACK1 is a major ceritinib target in MDA-MB-453

**Fig. 1:**
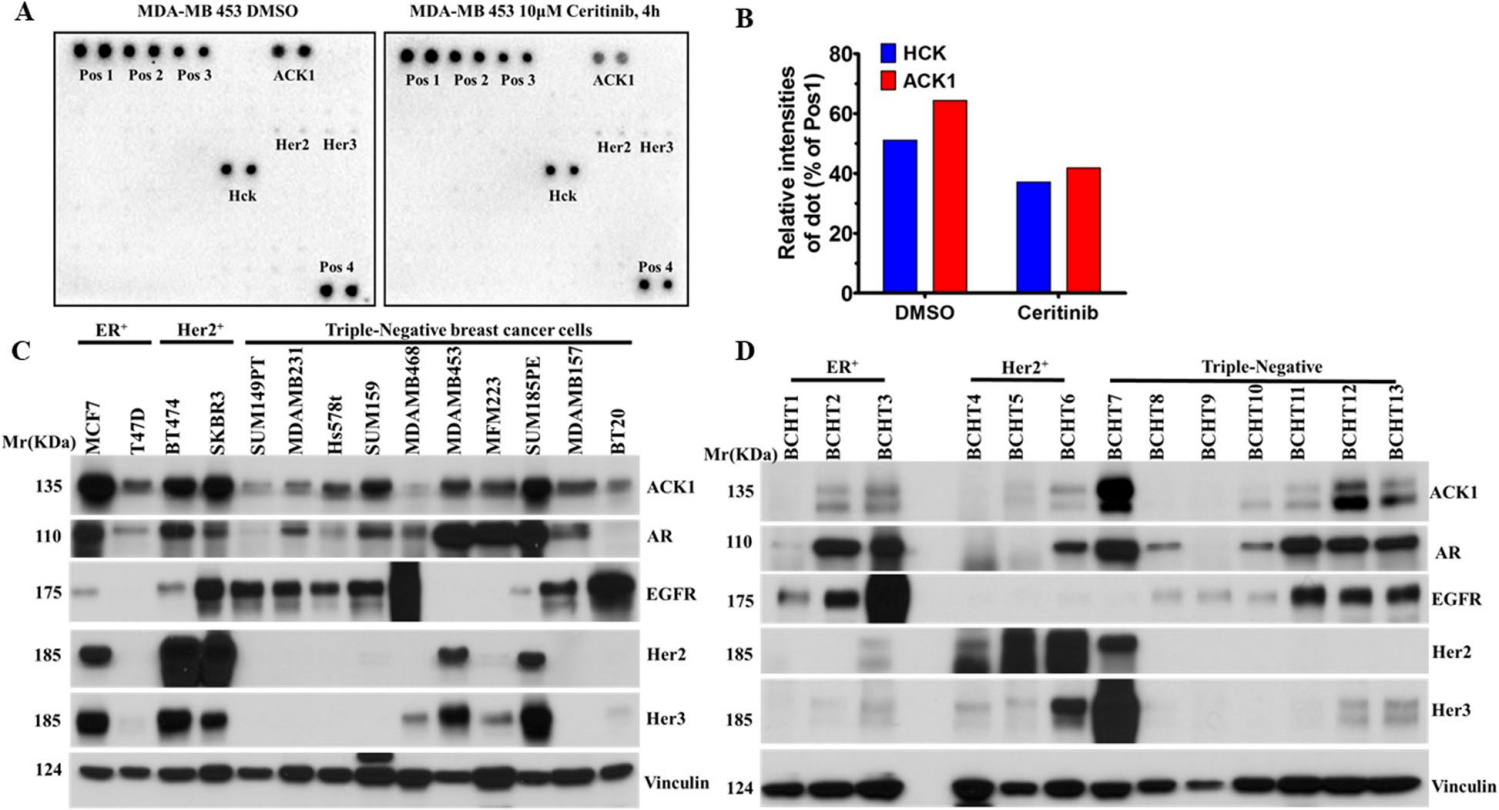
ACK1 is a major Ceritinib target in MDA-MB 453. (**A**) The levels of phosphorylated tyrosine kinases in a panel of 73 RTKs were analyzed by a human RTK Phosphorylation Antibody Array C1 Kit (RayBiotech) after MDA-MB 453 cells were treated with 10 μM ceritinib, DMSO was used as control. The positive dot#1,#2,#3 are all controlled amount of biotinylated antibody, which is consistent from array to array. As such, the intensity of these Positive Control signals serves much like housekeeping proteins. (**B**) The densitometric values of phospho-RTKs were determined by an image lab software (Bio-Rad). The relative intensities of the duplicated spots were normalized to positive control 1 spots. Values represent the mean of duplicate spots for each protein after normalization. (**C**) AR and ACK1 were determined by WB assay in human breast cancer cell lines and (**D**) in human breast cancer tissues

Ceritinib exhibits activity against ALK, ROS1, IGF-1R, FAK, ACK1/TNK2 and many other tyrosine kinases [20–22]. To determine which RTKs targeted by ceritinib, we employed a RTK phosphorylation antibody array that contains several proteins [26]. We found that ACK1 is a major ceritinib target in MDA-MB-453 cells (Fig. 1 A and B). ACK1 is one of the major activated tyrosine kinases in aggressive TNBC cells according to a global phosphotyrosine proteasome study by John Hopkins University [27]. Another study also revealed ACK1 is a potential TNBC therapeutic target [24]. As a cytoplasmic kinase, ACK1 activates AR in prostate cancer [28]. However, it is not clear whether ACK1 and AR protein expression is associated in breast cancer. To explore the importance of ACK1 and AR in breast cancer, we analyzed their expression in a panel of 14 human breast cancer cell lines and 13 human breast cancer tissues by Western blot assay. We found 64% (9 of 14) and 57% (8 of 14) of cells expressed ACK1 and AR, respectively (Fig. 1 C). Similarly, 57 and 64% of human breast tumor tissues expressed ACK1 and AR, respectively (Fig. 1 D). The majority of breast cells and tissues tested concurrently expressed ACK1 and AR (Fig. 1 C and D) suggesting that there is a link between ACK1 and AR.in breast cancer.

### Ceritinib inhibits RTK-ACK1-AR signaling pathways

**Fig. 2:**
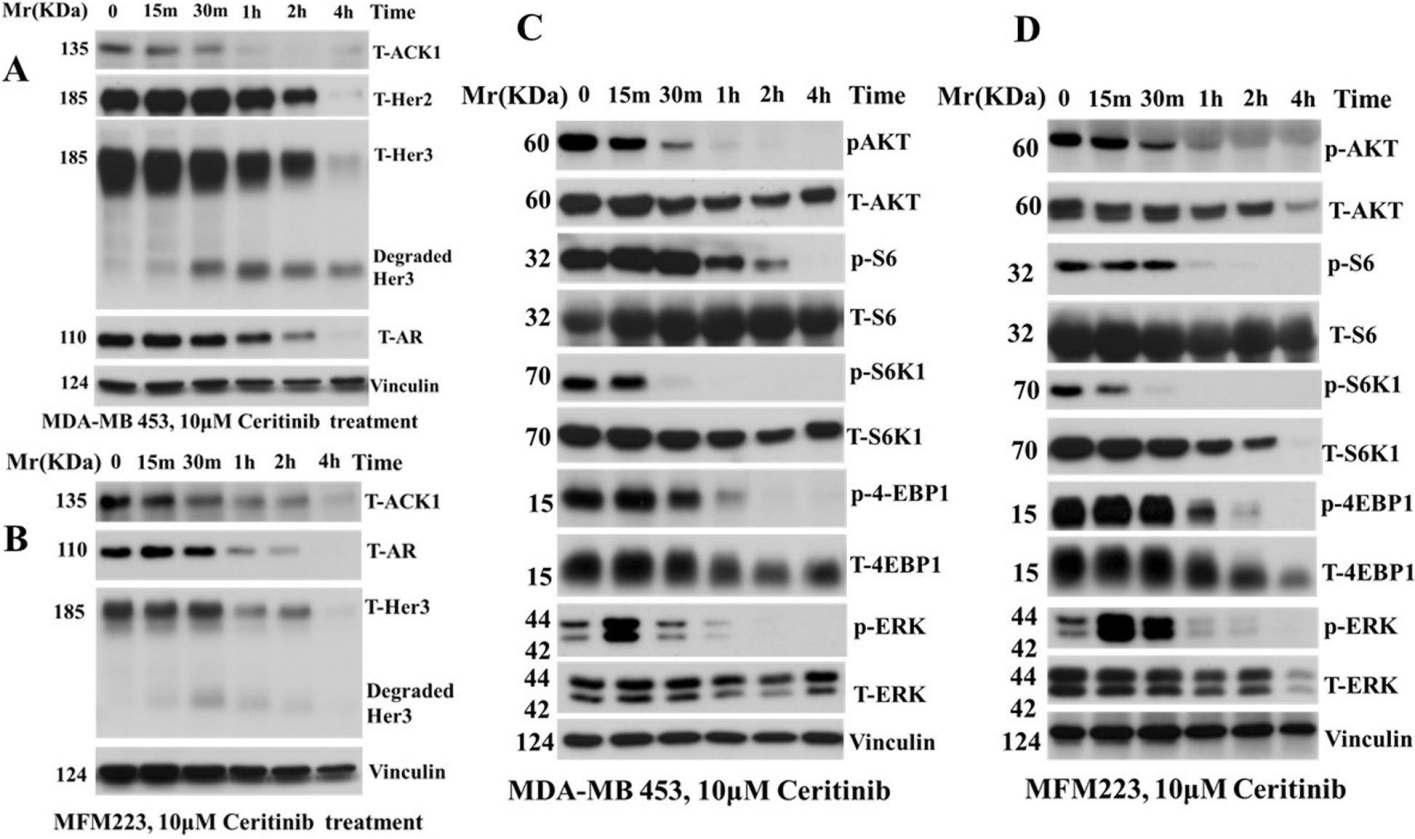
Delineation of molecular mechanisms of ceritinib effects on LAR killing. (**A**) Ceritinib treatment dramatically inhibited RTK-ACK1-AR signaling in time course in MDA-MB 453 cells and (**B**) MFM223 cells. Ceritinib treatment also dramatically suppressed AKT-mTOR signaling in MDA-MB 453 cells (**C**) and MFM223 cells (**D**) in time course. Cell skeleton protein vinculin was used as a protein loading control

Cell surface RTKs including IGF-IR, PDGFR, EGFR and HER3 are active in many TNBCs [29] (Fig. 1 C and D). RTKs can initiate androgen-independent AR signaling pathways, in which RTKs activate ACK1, which then forms a complex with AR and phosphorylates tyrosine residues in AR. Phosphorylated AR translocates to the nucleus with ACK1 and promotes transcription of oncogenes [28, 30]. To determine the importance of the RTK-ACK1-AR signaling axis as a possible mechanism for ceritinib-induced cytotoxicity in the AR^+^ TNBCs, we examined protein levels of AR, ACK1, HER2 and HER3 after ceritinib treatment in LAR cells. Ceritinib treatment downregulated AR, ACK1, HER2 and HER3 in MDA-MB-453 and MFM223 cells in a time-dependent manner (Fig. 2 A and B). For unknown reasons, Her3 is degraded. HER3 is a catalytically impaired pseudokinase. It is involved in the tumorigenesis in many kinds of cancers. HER3 could form powerful carcinogenic heterodimers with EGFR or HER2. Her2-Her3 heterodimers are potent signaling units. Even through MDA-MB 453 is considered a triple negative breast cancer, Her2 and HER3 are highly expressed in MDA-MB-453 [31]. Little is known about mechanisms of the Her3 downregulation from the plasma membrane, but there is evidence showing that Her3 is internalized and degraded in a clathrin-dependent manner [32]. The data suggested that ceritinib treatment inhibited RTK-ACK1-AR signaling pathways in LAR cells. In addition, activation of signaling pathways downstream of RTKs, including AKT-mTOR and MAPK cascades, are critical for TNBC tumorigenicity. Ceritinib treatment efficiently inhibited the phosphorylation of AKT, ERK1/2, S6, S6K1 and 4-EBP1, which are major players in AKT-mTOR and MAPK pathways (Fig. 2 C and D).

### Ceritinib inhibits WNT/β-catenin signaling pathway and cancer stem cell markers

**Fig. 3:**
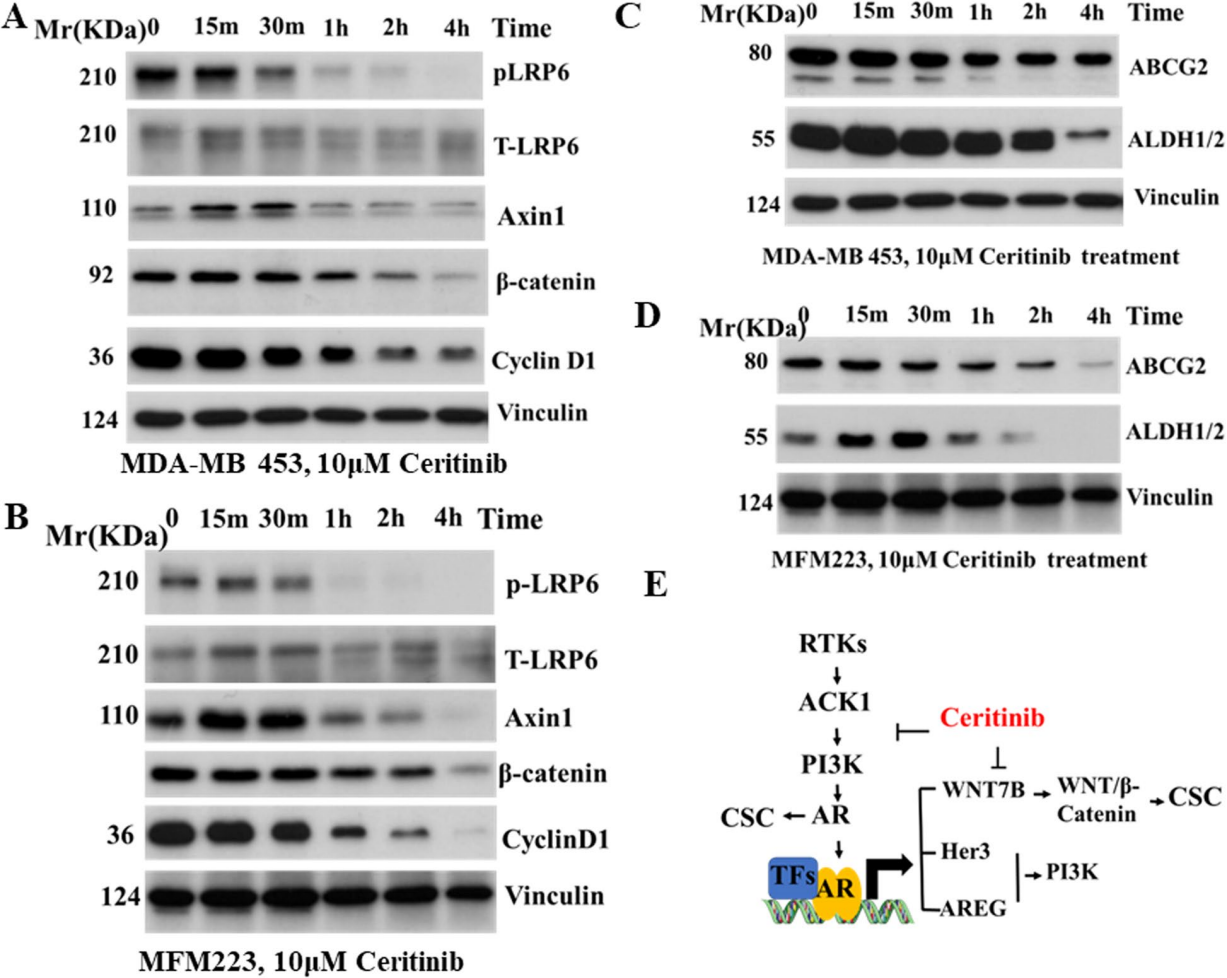
Ceritinib inhibits WNT/β-catenin signaling pathway and cancer stem cell markers. (**A**) Ceritinib treatment inhibited major WNT signaling proteins in time course in MDA-MB 453 cells and (**B**) MFM223 cells. Ceritinib treatment also significantly suppressed ABC transporters ABCG2 and stem marker ALDH1/2 in MDA-MB 453 cells (**C**) and MFM223 cells (**D**) in a time dependent manner. Vinculin was used as a protein loading control. (**E**) The proposed model

WNT deregulation is a major oncogenic driving force in TNBC. AR drives WNT and HER2 signaling pathways by transcriptional upregulation of WNT7B and HER3 [33]. We found that ceritinib treatment significantly suppressed the expression of proteins that are major players in the Wnt signaling pathway, including Axin1, LRP6 and β-catenin in multiple cell lines (Fig. 3 A and B). Cyclin D1 is a target gene of β-catenin. Ceritinib treatment suppressed the protein level of cyclin D1 (Fig. 3 A and B). Aberrant activation of Wnt signaling has been associated with the cancer stem cell (CSC) phenotype in breast cancer. AR maintains a CSC-population in TNBCs [34]. The existence of drug-resistant cancer stem cells (CSCs) is one of the main reasons why cancers are so difficult to treat effectively. Thus, we hypothesized that ceritinib treatment could overcome drug resistance through the presence of CSC populations by suppressing AR and Wnt signaling pathways. Indeed, we found that ceritinib treatment significantly decreased expression of the CSC marker ALDH1/2. In addition, multiple ABC transporters including ABCG2 enhance drug resistance in breast cancer. We found that ceritinib inhibited the expression of ABCG2 (Fig. 3 C and D). Together, these data indicate that ceritinib potentially inhibits the CSC population (Fig. 3 E) in LAR TNBC to overcome drug resistance in TNBC.

### A novel therapeutic strategy for AR^+^ TNBC tumors through the dual blockade of androgen-dependent and androgen-independent AR signaling pathways

**Fig. 4:**
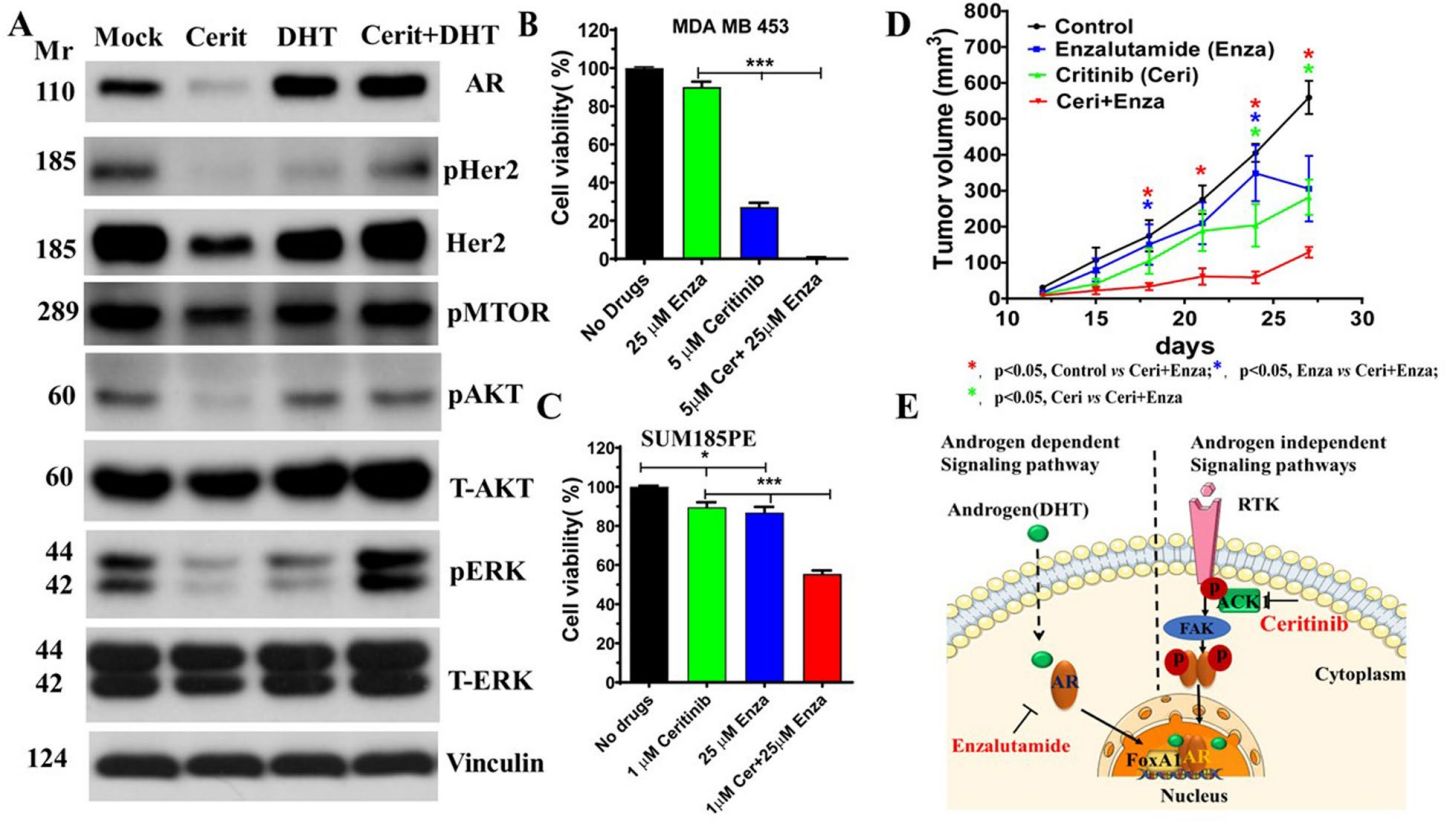
The combination of ceritinib and enzaluamide has greater effects on tumor-inhibition activity of LAR TNBCs. (**A**) The activation of androgen-dependent AR signaling compensated the inhibition of androgen-independent AR signaling pathways by ceritinib. MDA-MB 453 cells grew in DMEM with 10% FBS. The cells were treated with DMSO, 10 μM ceritinib, 100 nM DHT, and 10 μM ceritinib with 100 nM DHT for 8 h. The poteins were detected by WB assay. The combination of ceritinib and enzaluamide showed synergistic effects in killing MDA-MB 453 (**B**) and SUM185PE cells (**C**). SUM159PT SCID mouse xenografts. Mice were divided into four groups randomly, (1) untreated control, (2) ceritinib (50 mg/kg bodyweight) alone, (3) enzaluamide alone (25 mg/kg of bodyweight), and (4) ceritinib (50 mg/kg bodyweight) + enzaluamide (25 mg/kg of bodyweight). Ceritinib or enzaluamide alone showed modest inhibition of tumor growth. However, the combination of ceritinib and enzaluamide robust suppression of tumor progression. (**E**) Proposed mechanism of the dual blockade of AR signaling pathways through the combination treatment of enzalutamide and ceritinib. AR signaling pathways include an androgen-dependent canonical signaling pathway and androgen-independent signaling pathways. The data represent mean ± SEM and are ananlyzed by GraphPad prism 8 software. * *p <* 0.05

Enzalutamide blocks AR canonical signaling and significantly inhibits dihydrotestosterone (DHT)-driven growth of LAR cells [11]. To achieve a more effective treatment response, there is a strong clinical interest in developing new therapeutic strategies by combining AR antagonists with other targeted drugs. Our data show that enzalutamide inhibits cell growth of SUM185, MDA-MB-157, MDA-MB-231 and MDA-MB-468 (Suppl Fig. 5A, B, C, D). Current clinical trials examine several combinations of drugs including AR inhibitors plus PI3K-mTOR inhibitors and AR inhibitors plus CDK4/CDK6 inhibitors in advanced AR-positive TNBC tumors [14]. RTKs including IGF-IR, PDGFR, EGFR and HER3 are active in many TNBCs [14]. We found that ceritinib downregulated androgen-independent RTK-AR signaling (Fig. 2). However, ceritinib treatment did not inhibit RTK-AR signaling in the presence of DHT (Fig. 4 A), suggesting a cross-talk between the canonical AR signaling and androgen-independent AR signaling pathways. The activation of androgen-dependent AR signaling would compensate for the inhibition of androgen-independent AR signaling pathways. Therefore, we hypothesized that the combination of ceritinib and enzalutamide would provide enhanced effects on tumor growth inhibiting activity in AR^+^ TNBCs. To test the hypothesis, we treated MDA-MB-453 and SUM185PE LAR cells with ceritinib, enzalutamide, and the combination of ceritinib and enzalutamide compared to vehicle control. We found that the combination of ceritinib and enzalutamide has a robust inhibitory effect on cell growth of MDA-MB-453 and SUM185PE cells (Fig. 4 B and C). We then translated our in vitro findings to in vivo conditions and we established SUM159PT mouse xenograft models in female SCID mice (Jackson Laboratory) by subcutaneous injections as described previously [35]. Ceritinib or enzalutamide treatment alone showed only a modest inhibition of tumor growth. However, the combination of ceritinib and enzaluamide acted synergistically to suppress tumor progression with more robust inhibition (Fig. 4 D). We calculated the Bliss score for a block of ceritinib combination with enzalutamide by using a drug interaction reference model introduced by C. I. Bliss in 1939, we found that ceritinib and enzalutamide combination treatment synergistically suppressed the growth of AR^+^ TNBC cells MDA-MB 453 and SUM185PE. In addition, we also showed ceritinib and enzalutamide combination treatment synergistically suppressed AR^+^ TNBC SUM159PE growth (Suppl Fig. 15).

FAK is a non-receptor tyrosine kinase and is often overexpressed in prostate cancer. FAK can promote androgen-independent AR stimulation in prostate cancer. FAK is the downstream of ACK1 in breast cancer [36]. To understand the role of FAK and ACK, we treated MDA-MB-453 and SUM185PE cells with FAK specific inhibitor (PF-573228) and ACK1 specific inhibitor (AIM-100). We found that both FAK inhibitor and ACK1 inhibitor suppressed MDA-MB-453 and SUM185PE cell proliferation in a dose dependent manner, and the cells were more sensitive to FAK inhibitor than ACK1 inhibitor (Suppl Fig. 10 A and B). However, ceritinib in combination with FAK inhibitor but not ACK1 inhibitor suppressed MDA-MB-453 and SUM185PE cell proliferation in a significant synergistic manner compared to single agent treatment alone Suppl Fig. 11 and 12). Since we have seen a significant synergistic effect when we used ceritinib with FAK inhibitor (but not ACK1 inhibitor), we then checked for FAK, p-FAK, and FAK downstream targets, YB1, and p-YB1 in MDA-MB-453 and SUM185PE cells. Combination of Ceritinib with FAK inhibitor suppressed the p-FAK and p-YB1 suggesting an inhibitory mechanism when combining these drugs (Suppl Fig. 13 A and B).

Furthermore, our immunohistochemical analysis indicated that combination treatment had more robust inhibition of cell proliferation (Ki67), angiogenesis (CD31) and YB1 signaling (Suppl Fig. 7C). These data suggest the combination of enzalutamide and ceritinib may be used as a novel approach for treating AR^+^ TNBC patients (Fig. 4 E).

### Ceritinib inhibits FAK-YB-1 signaling pathways

**Fig. 5:**
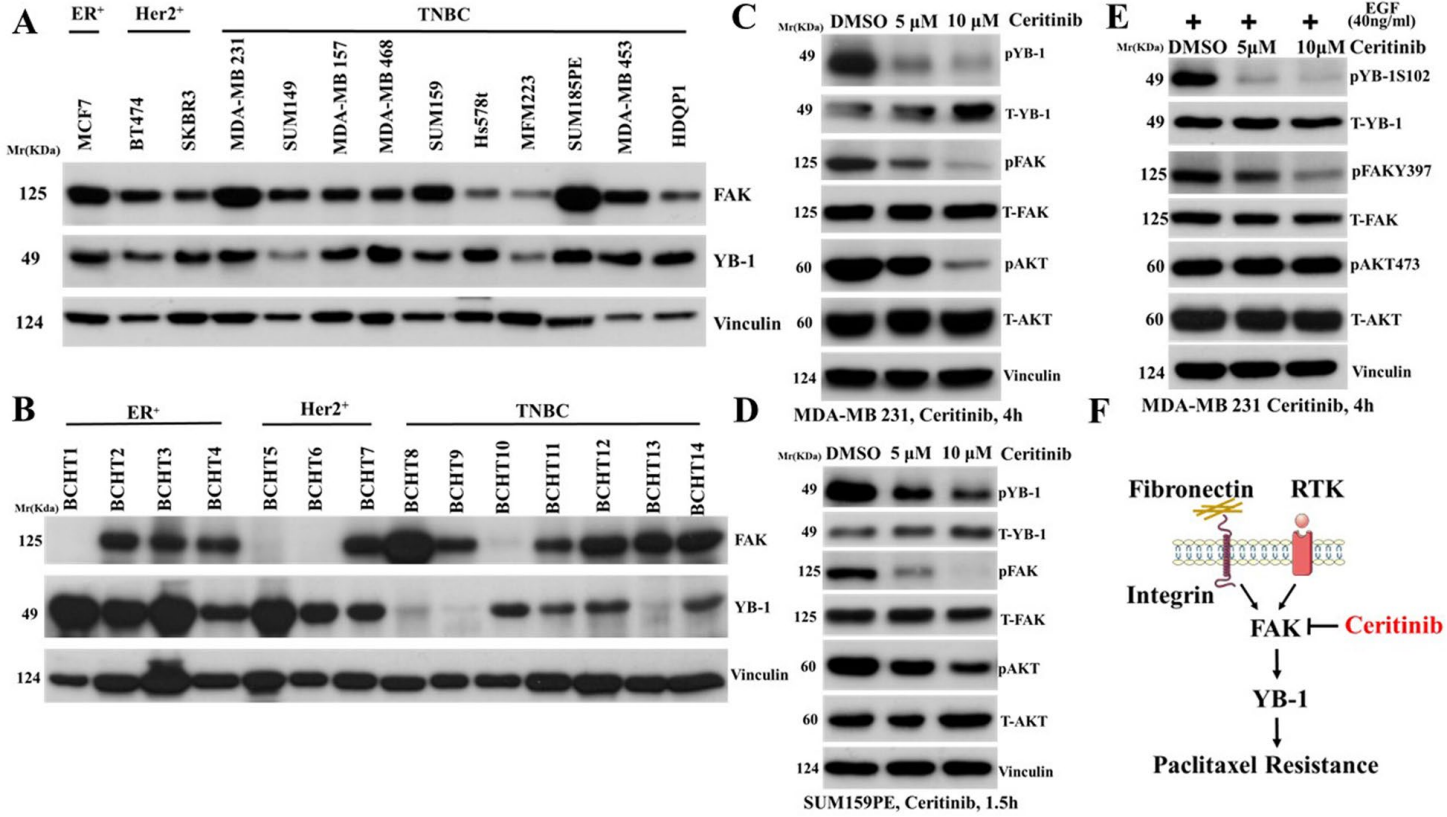
Ceritinib inhibits FAK-YB-1 signaling pathways. (**A**) The total protein level of FAK and YB-1 were determined by WB assay in human breast cancer cell lines and (**B**) in human breast cancer tissues, Vinculin was used as a protein loading control. (**C**) Ceritinib treatment inhibited FAK activity in MDA-MB 231 and (**D**) SUM159PE cells. Cells were grown in DMEM with 10% FBS overnight. The cells were then treated with DMSO, 5 μM and 10 μM ceritinib for 4 h. The poteins including YB-1, FAK, AKT and phosphorlated YB-1, FAK, AKT were detected by western blot assay. (**E**) MDA-MB 231 cells grown in DMEM containing 40 ng/ml EGF and 10% FBS. The cells were treated with DMSO, 5 μM and 10 μM ceritinib for 4 h. The total and phosphorlated YB-1, FAK, AKT proteins were detected by western blot assay. (**F**) The Proposed mechanism. FAK inhibition by ceritinib may overcome YB-1 mediated chmoresistance

We have proposed a combination therapeutic strategy for AR^+^ TNBCs. However, nearly half of TNBC patients do not have AR and many of them only express very low level of AR. It is possible that AR^−^ or AR-low TNBC tumors can also benefit therapeutically with ceritinib treatment. Paclitaxel (PTX) is an FDA approved chemotherapeutic agent in TNBC [37]. However, a substantial population of TNBC patients develop resistance to PTX therapy [38]. Thus, new therapeutic approaches are needed to overcome PTX resistance in TNBC treatment. Increased FAK expression is associated with metastasis and poor prognosis in breast cancer [25]. Inhibition of FAK is one proposed mechanism to overcome YB-1 mediated PTX resistance in ovarian and lung cancers [39]. As shown before, we found that ceritinib in combination with FAK inhibitor but not ACK1 inhibitor suppressed MDA-MB-453 and SUM185PE cell proliferation in a significant synergistic fashion compared to single agent treatments (supplementary Figs. 12 and 13). To explore the importance of FAK and YB-1 in breast cancer, we first analyzed their expression in a panel of 14 human breast cancer cell lines by western blot (WB) assay. We found that all human breast cancer cell lines expressed FAK and YB-1 (Fig. 5 A). In addition, we observed breast cancer patient tumors also express FAK and YB-1 (Fig. 5 B).

Upon integrin-fibronectin interaction or RTK activation by EGF, FAK is activated by tyrosine phosphorylation on a critical residue at 397 (Y397) (Supp Fig. 6 A). Ceritinib has been shown to inhibit FAK activity in melanoma and lung cancers [20, 21]. Consistent with this, we found that ceritinib significantly inhibited the FAK-YB-1 axis, as evidenced by decreased phosphorylation levels of proteins downstream of FAK activation including pFAK Y397, pAKT S473, pYB-1 S102 (Fig. 5 C, D and F, Sup Fig. 6B and C). Furthermore, ceritinib treatment inhibited FAK-YB1 activity in MDA-MB-231 cells upon EGF stimulation. (Fig. 5 E and F).

**Fig. 6:**
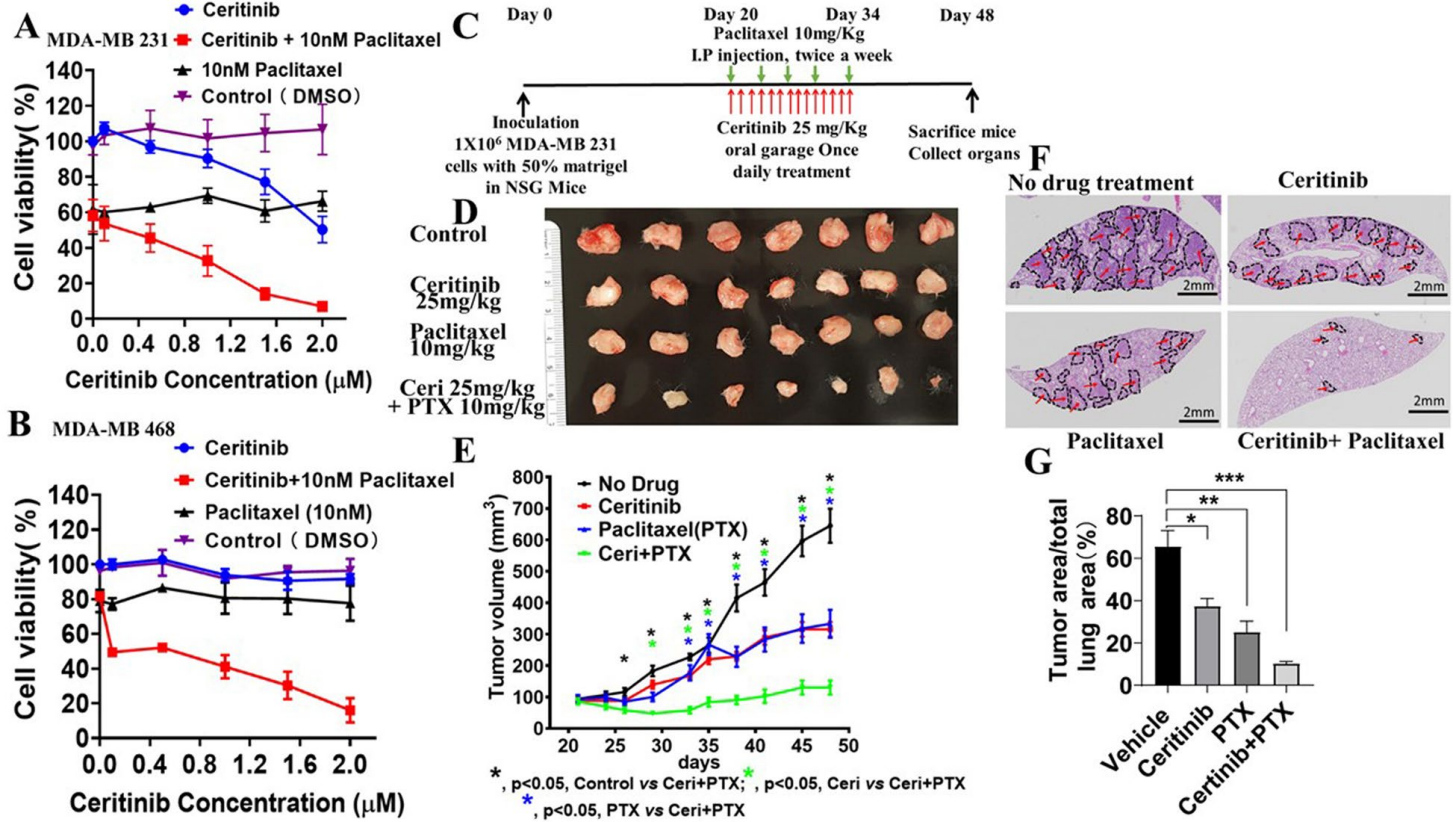
Combination treatment of ceritinib and paclitaxel (PTX) has great inhibitory effects on AR^−^ or AR^low^ TNBC cells growth in vitro and in vivo. Effect of ceritinib + paclitaxel on cell viability of MDA-MB 231 Cells (**A**) and MDA-MB 468 cells (**B**). Cells were grown in DMEM with 10% FBS overnight. The cells were then treated with different dosages of ceritinib with or without 10 nM paclitaxel for 72 h. The cell viability was determined by MTT assays. MDA-MB-231 cells 231 xenograft mouse model was established in 4-5 week-old NSG female mice. Mice were assigned into four groups randomly, which were untreated control, ceritinib (25 mg/kg bodyweight) alone, PTX alone (10 mg/kg of bodyweight), and ceritinib (25 mg/kg bodyweight) + PTX (10 mg/kg of bodyweight). The tumor volume and body weight were monitored twice a week. The combination of ceritinib and PTX synergistically suppressed the MDA-MB231 xenograft tumor growth. **C**, experimental plan; **D**, Different tumors, **E**, tumor volume measured over a period of 50 days. **F**, the lung metastases were located, the quantification results were generated using Image J software in **G**. (sections of lungs were stained with H&E)

### Combination treatment of ceritinib and paclitaxel strongly affects AR^−^ or AR^low^ TNBC growth in vitro and in vivo

The combination of FAK inhibition and paclitaxel has a strong effect in ovarian and lung cancers [21, 39]. Based on this, we hypothesized that inhibiting FAK by ceritinib will improve PTX-induced apoptosis in AR^−^ or AR^low^ TNBCs, and the combination of ceritinib and PTX will have an enhanced inhibitory effect on tumor cell growth. MDA-MB-231 and MDA-MB-468 cells are AR^low^ TNBC, which express high levels of FAK [40]. To test the hypothesis, we treated MDA-MB-231 and MDA-MB-468 cells with DMSO (control), ceritinib, PTX, and the combination of ceritinib and PTX, and found that the combination of ceritinib and PTX had a robust inhibitory effect on cell growth of MDA-MB 231 and MDA-MB 468 cells (Fig. 6 A and B). To further explore the therapeutic utility of the combinational therapy of ceritinib and PTX in vivo, we inoculated NSG mice with MDA-MB-231 cells. Strategy is shown in Fig. 6 C. Four treatment groups were included, (1) untreated control, (2) ceritinib (25 mg/kg bodyweight) alone, (3) PTX alone (10 mg/kg of bodyweight), and (4) ceritinib (25 mg/kg bodyweight) + PTX (10 mg/kg of bodyweight). Overall, ceritinib or PTX alone treatment decreased tumor progression. The combination of ceritinib and PTX showed robust inhibitory effect on the tumor growth and lung metastasis, compared to single drug alone (Fig. 6 D, E and F). These findings are partially due to a reduction in cell proliferation as demonstrated by Ki-67 staining of tumors (Supplementary Fig. 7A, B). To make our results more clinically relevant, we tested our hypothesis using the PDX model TU-BcX-4EA-LNb. We implanted intact tumor pieces coated in Matrigel™ in NSG mice and treated the tumors after they reached a designated volume in groups outlined in the previous xenograft experiment. We demonstrated TU-BcX-4EA-LNb to exhibit high expression of both AR and ACK1 (Supplementary Fig. 8). Baseline AR and ACK1 expression were also tested in other TNBC PDX models (Supplementary Fig. 8A). Two additional TNBC PDX models exhibited moderate expression of AR (TU-BcX-4 M4, TU-BcX-4IC) but to a dramatically lesser extent than TU-BcX-4EALNb. ACK1 expression was more variably expressed in the TNBC PDX tumors tested (Supplementary Fig. 8B).

**Fig. 7:**
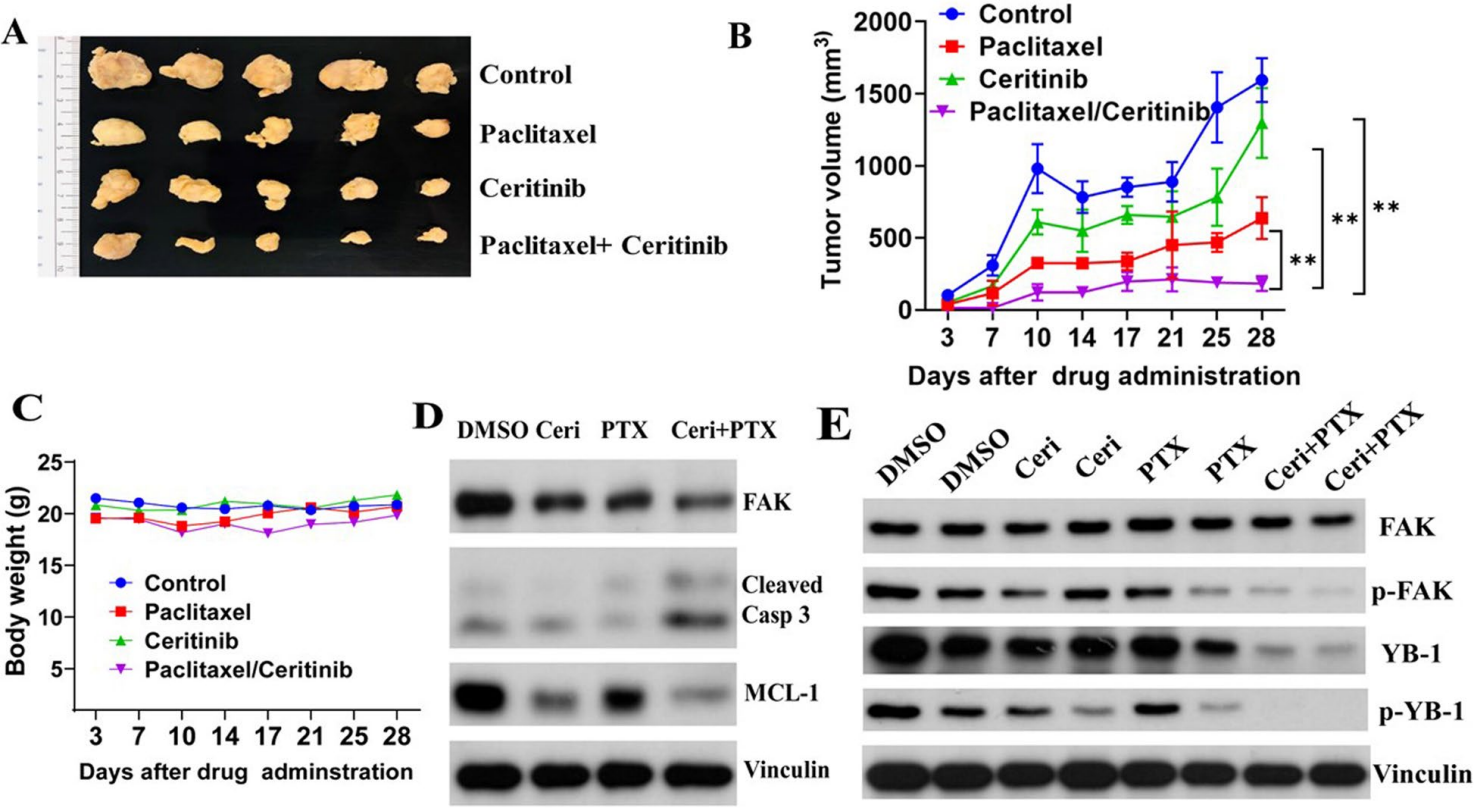
Combination treatment of ceritinib and paclitaxel (PTX) has robust inhibitory effect on patient derived xenograft (PDX) model. The combination of ceritinib and PTX synergistically suppressed the TU-BcX-4EALNb PDX tumor growth. The tumor volume and body weight were monitored twice a week. The combination of ceritinib and PTX synergistically suppressed the TU-BcX-4EALNb PDX tumor growth. TU-BcX-4EALNb tumors were implanted in 4-5 week-old NSG female mice. Mice were assigned into four groups including untreated control, ceritinib (25 mg/kg bodyweight) alone, PTX alone (10 mg/kg of bodyweight), and ceritinib (25 mg/kg bodyweight) + PTX (10 mg/kg of bodyweight). **A**, Different tumors; **B**, tumor volume measured over a period of 28 days; **C**, body weight measured over a period of 28 days. The data represent mean ± SEM and are ananlyzed by GraphPad prism 8 software. * *p <* 0.05; ** *p <* 0.01; *** *p <* 0.001. **D**, Ex vivo ceritinib and PTX combination treatment significantly increased the apoptosis in TU-BcX-4EALNb PDX xenograft tumors. The freshly isolated TU-BcX-4EALNb tumor was cut into 2 × 2 mm pieces, the tumor slices were treated with DMSO, 1 μM ceritinib, 10 nM PTX and 1 μM ceritinib + 10 nM PTX for 48 h. The apoptotic proteins were detected by western blot assay, and the downstream signaling proteins p-FAK and p-YB-1 were examined by western blot assays in **E**

Next we examined the effect using the TNBC PDX model that expressed AR and ACK1. Although ceritinib and PTX alone inhibited tumor growth, combination treatment with both drugs together much stronger inhibition of tumor growth (Fig. 7 A, B). Importantly, there was no change in body weight suggesting minimal toxic effects of the combination treatment (Fig. 7 C). Furthermore, ex vivo ceritinib and PTX combination treatment significantly increased apoptosis in PDX tumors as exhibited by increasing cleaved caspase 3 and reducing MCL1 (Fig. 7 D). To show the therapeutic outcomes and confirm the cellular mechanistic data, we isolated the fresh 4QATb2 PDX tumor and cut it to 3 × 3 mm^3^ small pieces, and treatment them with DMSO (control), ceritinib (Ceri), paclitaxel (PTX) and Ceri + PTX for 48 h, the treated samples were examined by western blot assays. We found that ceritinib and enzalutamide combination treatment significant suppressed the p-FAK and pYB-1 than single treatment (Fig. 7 E). These data strongly suggest that the combination of ceritinib and PTX is more effective than single drug alone in AR^−^ and AR ^low^ TNBC cancers.

In our study, we found that ceritinib inhibited the RTK-ACK1/FAK-AR axis in LAR TNBC cells. Consistent with this, data collected from public databases indicate that ACK (TNK2 gene) positively correlates with AR, Her3 (ERBB3), WNT signaling proteins such as Axin 1, β-catenin and cyclin D1 (Supplementary Fig. 9).

## Discussion

Advanced screening and imaging technology, effective chemotherapy, targeted therapy and immunotherapy have extended lives of breast cancer patients significantly. About 98% of patients with early-stage breast cancer can survive for 10–15 years or longer (https://www.breastcancer.org/symptoms/types/triple-negative). The five-year survival rate of breast cancer is about 90% in Western countries. TNBC is the most aggressive subtype of breast cancer and has the worst outcomes. The five-year overall survival of TNBCs is 78.5%. Even worse, the five-year survival rate for locally advanced and metastatic TNBC is only 11%. In the last decade, the success of PD-L1/PD-1 inhibitors, PARP inhibitors, and anti-Trop-2 antibody drug conjugates yielded several targeted treatment options for TNBC patients. Many TNBC tumors do not express biomarkers that would benefit from these recently FDA-approved targeted therapies (https://www.breastcancer.org/symptoms/types/triple-negative). Therefore, there are unmet medical needs to develop new targeted drugs or strategies for TNBC. Furthermore, acquired resistance to targeted therapies remains a large obstacle in developing effective therapeutics. Synergistic drug combinations are becoming promising new avenues to overcome predicted drug resistance.

There have been impressive advancements in targeted therapy development for TNBC. BRCA1 is a risk gene linked to TNBC. TNBC tumors carrying BRCA mutations, 10-15% of TNBC tumors, are sensitive to poly-ADP-ribose polymerase (PARP) inhibitor therapy [41]. Olaparib is an example of PARP1 inhibitor that has been shown to provide a significant benefit over standard chemotherapy for metastatic, germline BRCA mutated HER2-negative breast cancers [41]. Recently, Olaparib received FDA approval to treat advanced-stage TNBC with a BRCA1 or BRCA2 mutation [41]. In addition, PD-L1 is expressed in approximately 40% of TNBC tumors and TNBC-associated tumor stromal and infiltrating immune cells in the tumor microenvironment [42]. Atezolizumab is an anti-PD-L1 monoclonal antibody. Adding atezolizumab to nab-paclitaxel chemotherapy significantly improved PD-L1 positive TNBC cohort median progression-free survival and a longer overall survival [43]. Atezolizumab in combination with the albumin-bound paclitaxel or nab-paclitaxel was recently approved to treat advanced or metastatic PD-L1-positive TNBC [43]. The success of PD-L1/PD-1 inhibitors, PARP inhibitors, and anti-Trop-2 antibody drug conjugates, demonstrates that targeted drugs have important therapeutic implications in TNBC but warrant further clinical consideration for the treatment of specific subsets of TNBC patients [44]. Despite these impressive clinical successes, 60% of TNBC patients’ tumors do not express PD-L1 and about 20% of TNBC patients carry germline BRCA1/2 mutations [45]. As a result, many TNBC patients would not benefit from these recently FDA-approved targeted therapies. It is known that 40% of TNBC patients develop metastatic disease and mortality from the cancer [2]. Therefore, there are unmet medical needs to develop targeted drugs for TNBCs.

Luminal androgen receptor (LAR) represents a subtype of TNBC characterized by the presence of AR signaling. Asian patients with TNBC tumors have a higher prevalence of the LAR subtype (23% in Chinese compared to 12% in White cohorts) [46]. Androgens activate the AR and commonly thought as male hormones [47], but they are detectable in the circulation of women [48]. The level of circulated testosterone in women is about 5% of those observed in men [49, 50]. AR^+^ TNBC patients hardly benefit from current standard chemotherapy regimens. Therefore, discovery of novel targeted and combination therapeutic approaches for this subset of tumors are of great clinical relevance. For example, PIK3CA mutations are highly prevalent in TNBC LAR subtype. The combination of AR antagonist bicalutamide with PI3K inhibitor GDC-0941 or the dual PI3K/mTOR inhibitor GDC-0980 reduced LAR xenograft tumor growth [51].

AR stimulates tumor development and progression in ER^−^ breast cancers [12, 13]. AR signal transduction pathways include the canonical AR signaling and androgen-independent AR signaling pathways. In the canonical AR signaling pathway, androgens such as DHT cross the cell membrane and drive target gene transcription [52]. In addition, RTKs can initiate androgen-independent ACK1-AR signaling pathways. This androgen-independent AR signaling does not respond to classic AR antagonists including bicalutamide and enzalutamide. RTKs activate an intracellular tyrosine kinase ACK1 and promote oncogene transcription [28, 30]. RTK-activated androgen-independent AR signaling may partially complement or compete with canonical AR signaling activity, which results in only modest efficacy of AR antagonism in AR positive TNBC treatments. Current AR-targeting studies focus on the blockade of the canonical AR signaling pathway, while the role of androgen independent AR signaling pathway in TNBCs is neglected. We proposed here that the canonical AR signaling and androgen independent AR signaling pathways should be blocked concordantly to improve the response of AR antagonist in AR^+^ TNBC. We further showed that the combination strategy inhibits drug resistance through suppression of the CSC phenotype.

Ceritinib is an ALK inhibitor approved by the FDA for the treatment of ALK^+^ metastatic non–small-cell lung cancer (NSCLC) in 2014 [53]. In our report, we provide evidence for a mechanism through which ceritinib exerts its effects. We identified Activated CDC42 Kinase 1 (ACK1) as a major ceritinib target in LAR TNBC cells and demonstrated that ceritinib inhibited the RTK-ACK1-AR axis in LAR TNBC cells. Consistent with this, ACK expression positively correlated with AR, Her3 (ERBB3), and WNT signaling proteins such as Axin 1, β-catenin and cyclin D1.

Due to the observed inhibitory effects of ceritinib in the AR signaling cascade, we proposed that an AR inhibitor and ceritinib would be synergistic and more effective in a combination strategy. In this study, we discovered that the combination of enzalutmide and ceritinib is an effective regimen in AR^+^ TNBC patients and the combination of PTX and ceritinib is an effective therapeutic strategy in AR^−^ and AR^low^ TNBC. The efficacy of these combination strategies were evaluated in multiple model systems. Furthermore, the diverse breast cancer cell lines and tumor specimens in our study addressed any potential cell line specific effects of the proposed combination drug strategies. We demonstrated these novel drug combinations were effective in a highly translational model system using intact patient-derived xenografted tumors, indicating our observations are translatable to the clinical setting. Furthermore, although paclitaxel is a commonly used therapeutic in breast cancer systemic chemotherapy regimens, resistance to paclitaxel results in tumor recurrence and drug resistance, limiting efficacy of paclitaxel-based strategies. In our study, we revealed ceritinib to inhibit FAK and YB-1 mediated mechanisms that contribute to paclitaxel resistance.

This study has crucial implications in the field of drug and target discovery in the context of breast cancer. The results from our study have immediate implications for clinical translation for this difficult-to-treat breast cancer subtype, which would benefit significantly from effective targeted therapeutic regimens. Our innovative approach to test repurposed FDA-approved drugs in new clinical contexts can be applied to all cancer tumor types and is not limited to breast cancer.

## Methods

### Experimental animals

All the mice were housed in a pathogen-free animal room under standard conditions with free access to water and food (standard chow diet and water ad libitum). All of the procedures were approved by the Institutional Animal Care and Use Committee of the LSU Health Science Center at New Orleans.

### Antibodies, cell lines and chemicals

MDA-MB-231, MCF7, T47D, SKBR3, BT474, BT-20, MDA-MB-468, Hs578t and MDA-MB-453 and MDA-MB-157 cells purchased from the American Type Culture Collection (Manassas, VA). MFM 223, SUM159PE and SUM185PE cells were kindly provided by Dr. Jennifer A. Pietenpol (Vanderbilt-Ingram Cancer Center). ACK1, AR, EGFR, Her2, Her3, Mcl-1, Bcl-2, Puma, YB-1, FAK, AKT, Anti-phospho-AKT (Ser473), anti-phospho-S6K1 (S79), Anti-phospho-4-EBP1, Anti-phospho-LRP6, Anti-phospho-mTOR, Anti-phospho-YB-1, Anti-phospho-FAK (Y397), anti- ribosomal protein S6, anti-ribosomal protein S6, BCL-xL and anti-cleaved Casp3 antibodies were obtained from Cell Signaling Technology (Beverly, MA). Anti-phospho-ribosomal protein S6 (S235/236), mouse anti-ERK antibody and Anti-phospho-ERK were purchased from Santa Cruz Biotechnology (Santa Cruz, CA). Anti-Vinculin was purchased from Sigma. Secondary anti-mouse IgG with horseradish peroxidase was from Calbiochem. Secondary anti-rabbit IgG with horseradish peroxidase was from GE Healthcare. Ceritinib and Paclitaxel were purchased from APExBIO. Bicalutamide (97%) purchased from ACROS Organics. The 133 FDA-approved drugs were kindly provided from NCI/DTP Approved Oncology Drugs Plated Set (AODVIII).

### Phospho-RTK Array analysis

To determine which receptor tyrosine kinases (RTKs) are targeted by ceritinib, the Human RTK Phosphorylation Antibody Array C1 Kit (RayBiotech, Cat. No. AAH-PRTK-1-2) was used. MDA-MB-453 cells were grown in DMEM medium containing 10% fetal bovine serum (FBS) and penicillin/streptomycin. When cells reached 70 ~ 80% confluence, media were changed to DMEM medium supplemented with DMSO, or 10 μM ceritinib. After 4 h incubation, cell lysates were prepared using lysis buffer containing protease and phosphatase inhibitors. After blocking for 1 hour at room temperature, the array membranes incubated with 700 μg of protein lysate overnight at 4 °C. Next, the arrays washed and incubated with biotin-conjugated phospho-tyrosine detection antibody overnight at 4 °C. Finally, the arrays incubated with a horseradish peroxidase-conjugated streptavidin (1:1000) at room temperature for 2 h. The phosphorylated RTKs detected and captured by ChemiDoc™ XRS+ Imaging Systems (Bio-Rad). The densitometric values of phospho-RTKs determined by an image lab software (Bio-Rad). The relative intensities of the duplicated spots were normalized to positive control spots. Values represent the mean of duplicate spots for each protein after normalization.

### Drug treatments for TNBC cells

Tumor cells were seeded onto 6-well plates at a density of 300,000 cells per well. After culturing in complete DMEM medium for 16 hours, media was replaced with fresh DMEM containing 10% FBS and 10 μM ceritinib for 4 hours. In control conditions, media was replaced with fresh DMEM containing 10% FBS. Cells at indicated time points were lysed in radioimmunoprecipitation (RIPA) lysis buffer with protease inhibitor cocktail (Roche) and phosphatase inhibitors. Protein concentration was determined using a bicinchoninic acid (BCA) protein assay (Thermo, Waltham, MA). Samples were boiled in SDS sample buffer for 10 minutes and stored at − 80 °C until analysis.

### Cell viability assay

TNBC cells were seeded (3000–5000 cells per well) in 96-well plates. Growth medium was replaced with either fresh medium (DMSO as a control) or medium containing the drugs after overnight growth. Cell viability was determined in quadruplicate using the MTT (3-(4,5-dimethylthiazol-2-yl)-2,5-diphenyltetrazolium bromide) assay, a colorimetric assay according to the conversion of tetrazolium salts to blue formazan products by active cells, in 96-well plates at the indicated time points. The replicates normalized to the control wells. To further confirm the results of MTT assay, the experiments were repeated using Cell Counting Kit 8 (WST-8 / CCK8) (Abcam, ab228554). The data analysis performed using Prism software (GraphPad Software). Data represent the mean ± SEM. Student’s t test was used to analyze the data, and a *p*-value of < 0.05 was considered statistically significant. * *P* < 0.05; ** *P* < 0.01, *** *P* < 0.0001.

### Ki 67 staining

Tissue sections were deparaffinized with xylene and re-hydrated through descending grades of alcohol up to water. Then, non-enzymatic antigen retrieval in Citrate buffer, pH 6.0 for 30 minutes at 95 °C, and endogenous peroxidase quenching with H2O2 in Methanol for 30 minutes were done. Sections were washed three times in PBS at interval of 10 minutes before blocking with 5% normal goat serum in 0.1% PBS/BSA. Tissues were incubated with Anti-Ki67 antibody (1:100 dilution; Cat# ab833, Abcam) overnight. After PBS washing, sections were incubated with a biotinylated anti-rabbit secondary IgG for 60 minutes, incubated with avidin-biotin-peroxidase (ABC) complexes, and developed with diaminobenzidine (Sigma).

### Analysis of MDA-MB 231 xenograft tumor development and metastasis

MDA-MB 231 (1 × 10^6^ cells) with 50% Matrigel™ were injected into Number 4 mammary gland of 4-5 weeks old NSG (Jackson Laboratory). Tumors were measured by a caliper. When tumors reached a size of ∼50 mm^3^ the mice were randomly distributed into four groups (seven mice in each group): (1) untreated control, (2) ceritinib (25 mg/kg bodyweight) alone, (3) paclitaxel (PTX) alone (10 mg/kg of bodyweight), and (4) ceritinib (25 mg/kg of bodyweight) + PTX (10 mg/kg of bodyweight). Ceritinib was dissolved in sesame oil and administrated by oral gavage once daily for 2 weeks. PTX (10 mg/Kg) was injected intra-peritoneally twice a week. Tumor volume measured twice a week after the initial injection, and the volumes were calculated using the formula (π x length x width1 x width2 /6). Mice were euthanized when they became moribund, or when they lost 20% weight. All organs examined for the presence of tumors and metastases at autopsy. Metastatic tumors were defined as nodules identified at secondary sites with a diameter ≥ 0.2 mm. To examine whether lungs infiltrated by metastases, lungs were fixed in formalin and embedded in paraffin. Sections stained with hematoxylin & eosin (H&E) staining. Data grouped and plotted using GraphPad Prism 8.

### Enzalutamide and ceritinib treatment for SUM159 xenograft mice

SUM159PE (5 × 10^6^ cells) with 50% Matrigel™ were injected into Number 4 mammary glands of 4-5 week old SCID mice (Jackson Laboratory). Tumors were measured by a caliper. When tumors reached ∼100 mm^3^ the mice were randomly distributed into four groups that included seven mice in each group: untreated control, ceritinib (50 mg/kg bodyweight) alone, enzalutamide alone (25 mg/kg of bodyweight), and ceritinib (50 mg/kg bodyweight) + enzalutamide (25 mg/kg of bodyweight). Drugs were administrated by oral garage once daily for 2 weeks. Tumor volume was measured twice a week after the initial injection. All the mouse experiments were performed in accordance with procedures and guidelines approved by Institutional Animal Care and Use Committee of the LSU Health Science Center at New Orleans.

### Patient-derived xenografts

The AR-positive triple negative patient-derived tumor, designated as TU-4EA-LNb, was derived from a metastatic lymph node after mastectomy surgery with lymph node dissection from an African-American patient. TU-4EA-LNb was established in 4–6 week old SCID/Beige (CB17.Cg-PrkdcscidLyst bg/Crl) mice provided from Jackson Laboratory, a mouse type chosen to optimize tumor take. Tumor volume was measured using a digital caliper. Intact tumor pieces (3 × 3 mm^2^) were coated with Matrigel™ (BD Biosciences) and implanted unilaterally in the mammary fat pads of SCID/Beige mice. When tumors reached ∼100 mm^3^ the mice were randomly distributed into four groups: (1) untreated control, (2) ceritinib (50 mg/kg bodyweight) alone, (3) enzalutamide alone (25 mg/kg of bodyweight), and (4) ceritinib (50 mg/kg bodyweight) + enzalutamide (25 mg/kg of bodyweight). Drugs were administrated by oral gavage once daily for 2 weeks. Tumor volume was measured twice a week after the initial injection. For ex vivo analysis, TU-4EA-LNb tumor pieces were plated in cell culture dishes and covered in DMEM containing 10% FBS and drug treatments or vehicle controls. After the designated treatment time explants were collected, mechanically disrupted using scissors, and enzymatically digested using Trizol. Tumor pieces of additional PDX models established by the Burow lab were utilized to evaluate baseline expression of AR and ACK1: TU-BcX-2 K1 (T8, T11), TU-BcX-4 M4 (Tb2, Tb3), TU-BcX-4QX (Tb1, Tb2), TU-BcX-4IC (T1, T3) and TU-BcX-4EA-LNb (passage 4). The number following ‘T’ in the nomenclature of the PDX models after ‘TU-BcX-‘denotes the number of times the tumor had been serially transplanted in mice before the tumor was removed for analysis. All the mouse experiments performed in accordance with procedures and guidelines approved by Institutional Animal Care and Use Committee of the LSU Health Science Center at New Orleans. PDX tissues were procured through the Louisiana Cancer Research Consortium Biospecimen Core and processed following NIH regulations and institutional guidelines of Tulane University with IRB exemption status. All animal procedures were conducted in compliance with State and Federal laws, standards of the U.S. Department of Health and Human Services, and guidelines established by the Tulane University Animal Care and Use Committee. The facilities and laboratory animals programs of Tulane University are accredited by the Association for the Assessment and Accreditation of Laboratory Animal Care.

### Mouse tumor tissue protein analysis

Fresh isolated tumor samples were snap frozen in liquid nitrogen. The extracts were prepared by grinding tissue into a fine powder in liquid nitrogen and subsequently dissolved in modified RIPA buffer supplemented with protease and phosphatase inhibitors. The lysates cleared by centrifuge at 13,000 rpm for 15 min and the supernatant was collected as extracted protein. Protein concentrations were determined using BCA protein assay. Equal amount of protein lysates separated by SDS-PAGE and then transferred to a PVDF membrane and detected with various antibodies.

### Statistical analysis

Data are shown as means ± SEM if not otherwise indicated. Two-tailed unpaired Student’s t-test was applied for statistical analysis to compare the two groups of interest and *P <* 0.05 was considered statistically significant unless otherwise stated. Graphical information performed using GraphPad Prism software (GraphPad Software Inc., San Diego, CA).

## Supplementary Information

**Additional file 1: SFig. 1.** Identification of FDA approved cancer specific drugs with single-agent activity against AR+ TNBCs. A Overview of the workflow of the drug screen. MDA MB 453 cells were treated with each drug (10 μmol/L) for 48 hours and cell viability was assessed using MTT assay. B Responses of the 133 drugs in NCI/DTP Approved Oncology Drugs Plated Set (AODVIII). Data show the inhibition of MDA-MB-453 growth at 10 μmol/L of drug relative to vehicle. C Detailed list of the drugs that results in < 50% of cell viability.

**Additional file 2: SFig. 2.** Ceritinib treatment efficiently killed LAR cells. Ceritinib killed MDA-MB-453 (A) and MFM223 cells (B) in a dose- and time-dependent manner. (C) The IC_50_ of ceritinib appeared to be 1.19 μM for MDA-MB 453 cells and 1.235 for MFM223 cells (D).

**Additional file 3: SFig. 3.** Ceritinib treatment efficiently killed LAR cells. A Ceritinib treatment inhibited MDA-MD-231, MDA-MB-453 and SUM185PE colony formation. B The number of colonies n in (B) were manually quantitated and plotted using GraphPad prism 8 software.

**Additional file 4: SFig. 4.** Ceritinib treatment efficiently killed multiple TNBC cells in a dosage-dependent manner. SUM185PE (A), MDA-MB 157 (B), MDA-MB 231(C) and MDA-MB 468 cells were treated with different concentration of ceritinib for 72 h. The cell viability was determined by MTT assay.

**Additional file 5: SFig. 5.** Ceritinib treatment efficiently killed multiple TNBC cells. SUM185PE (A), MDA-MB 157 (B), MDA-MB 231(C) and MDA-MB 468 cells were treated with different concentration of ceritinib for 72 h. The cell viability was determined by Cell Counting Kit 8 (WST-8 / CCK8) (Abcam, ab228554).

**Additional file 6: SFig. 6.** Ceritinib inhibits FAK-YB-1 signaling pathways. A Ceritinib treatment inhibited FAK and YB-1 activities in MDA-MB 231 cells. Cells grew in DMEM containing 10% FBS with or without 40 ng/ml EGF, or with or with 20 ng/ml fibronectin (FN) for 4 h. The total and phosphorlated YB-1, FAK, AKT proteins were detected by western blot assay. Ceritinib treatment inhibited FAK-YB-1 signaling in SUM185PE (B) and MDA-MB 468 cells (C).

**Additional file 7: SFig. 7.** Combination of Ceritinib and paclitaxel inhibits cell proliferation in tumors Cell proliferation of tumors derived from MDA MB 231 cells treated with drugs. A Paraffin-embedded tissue sections of primary tumors from mice were immunostained with anti-Ki67 antibody Photomicrographs were taken at 40× magnification; scale bars = 100 μM. B Quantification of cell proliferation in tumors treated with drugs. The number of Ki67-positive cells per field is shown as an average of three of fields viewed. C Paraffin-embedded tissue sections of primary tumors from mice treated with Ceritinib, Enzalutamide or combination were immunostained with anti-Ki67, CD31, and P-YB1 antibodies. D The number of antibody-positive cells per field is shown as an average of three of fields viewed. Data are given as mean ± SD. Statistically significant values of *p < 0.05, **p < 0.01, and ***p < 0.001 were determined compared with the control. Scale bars = 100 μM.

**Additional file 8: S Fig. 8.** PDX models. A Characteristics of PDX models (B) Expression of AR and ACK1 in PDX models.

**Additional file 9: S Fig. 9.** Analysis of proteins involved in AR signaling pathway. Correlation analysis of the METABRIC breast cancer data set comparing the correlation between ACK (TNK2):AR expression (mRNA expression Z-scores) ratios. The Pearson correlation coefficients (r) and the relative *p*-values are shown. The association between genes was measured using the Pearson correlation coefficient (r) and respective computed *P*-value. Using cBioPortal, analyzed the co-expression of TNK2 mRNA expression in breast cancer patients. There were co-expression correlations between the following genes: TKN2 positively correlates with ERBB3, AXIN1, CCND1, and PTK2 (FAK).

**Additional file 10: S Fig. 10.** PF-573228 and AIM-100 inhibited TNBC cell growth. A The effects of the FAK inhibitor (PF-573228) on MDA-MB-453 and SUM185PE proliferation were estimated by MTT assay after 24 h of treatment. IC50 values were collected from three independent experiments, each performed in triplicate. IC50 is the concentration of drug that caused a 50% reduction in proliferation compared to the vehicle-treated cells. B The effects of the ACK1 inhibitor (AIM-100) on MDA-MB-453 and SUM185PE proliferation were estimated by MTT assay after 24 h of treatment. Data are given as mean ± SD. Statistically significant values of *p < 0.05.

**Additional file 11: S Fig. 11.** ACK inhibitor AIM-100 did not have a synergistic effect with ceritinib. Examined in two different cell lines (MDA-MB-463 and SUM 185PE).

**Additional file 12: S. Fig. 12.** FAK blockade increased ceritinib sensitivity in MDA-MB-453 and SUM185PE cells. A The effects of PF-573228-ceritinib therapy on cell proliferation were investigated by MTT assay. The cultures were treated with PF-573228 (5 μM) and ceritinib (2.5 and 5 μM) for 24 h. B The 2D Bliss synergy map confirmed the synergistic effect of the PF-573228-ceritinib combination. The cell cultures were treated with ceritinib (2.5 and 5 μM) and PF-573228 (5 μM) for 24 h. 2D synergy map of PF-573228-ceritinib combination calculated by Bliss method showed the synergistic combination of PF-573228 and ceritinib. Bliss scores more than 10 located indicate a synergistic drug-drug interaction. C The effects of PF-573228-ceritinib combination therapy on cell viability were demonstrated by crystal violet staining. The cells were treated with the PF-573228 and ceritinib inhibitors for 24 h, then stained with crystal violet and imaged by an inverted microscope (images acquired at 10x magnification). Data are given as mean ± SD. Statistically significant values of ***p < 0.001 and ****p < 0.0001 were determined compared with the control **p < 0.01 were determined compared with the control.

**Additional file 13: S Fig. 13.** PF-573228-ceritinib combination inhibits the FAK pathway. The effects of A ceritinib (2.5 μM), PF-573228 (5 μM), PF-573228-ceritinib (B) AIM-100 (5 μM), ceritinib (2.5 μM), and AIM-100-ceritinib combination on FAK and ACK1 pathway and their down-stream targets were determined by Western blot analysis in MDA-MB-453 and SUM185PE cells.

**Additional file 14: S Fig. 14.** Combined treatment with ceritinib and paclitaxel results in anti-proliferative synergism. A The effects of ceritinib-paclitaxel therapy on cell proliferation were investigated by MTT assay. The cells were treated with paclitaxel (2.5 μM) and ceritinib (1, 2.5 and 5 μM) for 24 h. B The 2D Bliss synergy map confirmed the synergistic effect of the paclitaxel-ceritinib combination. The cell cultures were treated with ceritinib (2.5 and 5 μM) and paclitaxel (1, 2.5, 5 μM) for 24 h. 2D synergy map of paclitaxel-ceritinib combination calculated by Bliss method showed the synergistic combination of paclitaxel and ceritinib. Bliss scores more than 10 located indicate a synergistic drug-drug interaction. Data are given as mean ± SD. Statistically significant values of ****p < 0.0001 were determined compared with the control or single treatments.

**Additional file 15: S Fig. 15.** Combined treatment with ceritinib and enzalutamide results in anti-proliferative synergism. The 2D Bliss synergy map confirmed the synergistic effect of the paclitaxel-ceritinib combination. The cell cultures were treated as shown in Fig. 4 B and C. Bliss scores more than 10 located indicate a synergistic drug-drug interaction.

## Data Availability

All data generated during this study are included in this published article and its supplementary files.

## Abbreviations

TNBC: Triple negative breast cancer
ER: Estrogen receptor
PR: Progesterone receptor
AR: Androgen receptor
LAR: Luminal androgen receptor
CSC: Cancer stem cell
MAPK: mitogen activated protein kinase
IGF: Insulin growth factor
PDGFR: Platelet derived growth factor receptor
EGFR: Epidermal growth factor receptor
RTK: Receptor tyrosine kinase
FAK: Focal adhesion kinase
PTX: Paclitaxel
SCID: Severe combined immune-deficient
DMSO: Dimethyl sulfoxide
PARP: Poly-ADP-ribose polymerase
PBS: Phosphate buffered saline
BSA: Bovine serum albumin
FBS: Fetal bovine serum
PDX: Patient derived xenograft
FN: Fibronectin
NSG: Nod scid gamma

## References

[1] Cho B, Han Y, Lian M, Colditz GA, Weber JD, Ma C, et al. Evaluation of racial/ethnic differences in treatment and mortality among women with triple-negative breast Cancer. JAMA Oncol. 2021. 10.1001/jamaoncol.2021.1254 PMC8120441 33983438

[2] Boyle P. Triple-negative breast cancer: epidemiological considerations and recommendations. Ann Oncol. 2012;23 Suppl 6:vi7-12. 10.1093/annonc/mds187 23012306

[3] Mahtani R, Kittaneh M, Kalinsky K, Mamounas E, Badve S, Vogel C, et al. Breast Cancer therapy expert G: advances in therapeutic approaches for triple-negative breast Cancer. Clin Breast Cancer. 2020. 10.1016/j.clbc.2020.12.011 33781662

[4] Mi H, Gong C, Sulam J, Fertig EJ, Szalay AS, Jaffee EM, Stearns V, Emens LA, Cimino-Mathews AM, Popel AS (2020). Digital pathology analysis quantifies spatial heterogeneity of CD3, CD4, CD8, CD20, and FoxP3 immune markers in triple-negative breast Cancer. Front Physiol.

[5] Lehmann BD, Jovanovic B, Chen X, Estrada MV, Johnson KN, Shyr Y, Moses HL, Sanders ME, Pietenpol JA (2016). Refinement of triple-negative breast Cancer molecular subtypes: implications for neoadjuvant chemotherapy selection. PLoS One.

[6] Gucalp A, Tolaney S, Isakoff SJ, Ingle JN, Liu MC, Carey LA, Blackwell K, Rugo H, Nabell L, Forero A (2013). Phase II trial of bicalutamide in patients with androgen receptor-positive, estrogen receptor-negative metastatic breast Cancer. Clin Cancer Res.

[7] Traina TA, Miller K, Yardley DA, Eakle J, Schwartzberg LS, O'Shaughnessy J, Gradishar W, Schmid P, Winer E, Kelly C (2018). Enzalutamide for the treatment of androgen receptor-expressing triple-negative breast Cancer. J Clin Oncol.

[8] Farmer P, Bonnefoi H, Becette V, Tubiana-Hulin M, Fumoleau P, Larsimont D, Macgrogan G, Bergh J, Cameron D, Goldstein D (2005). Identification of molecular apocrine breast tumours by microarray analysis. Oncogene.

[9] Lehmann BD, Pietenpol JA (2014). Identification and use of biomarkers in treatment strategies for triple-negative breast cancer subtypes. J Pathol.

[10] Cochrane DR, Bernales S, Jacobsen BM, Cittelly DM, Howe EN, D'Amato NC, Spoelstra NS, Edgerton SM, Jean A, Guerrero J (2014). Role of the androgen receptor in breast cancer and preclinical analysis of enzalutamide. Breast Cancer Res.

[11] Barton VN, D'Amato NC, Gordon MA, Lind HT, Spoelstra NS, Babbs BL, Heinz RE, Elias A, Jedlicka P, Jacobsen BM (2015). Multiple molecular subtypes of triple-negative breast cancer critically rely on androgen receptor and respond to enzalutamide in vivo. Mol Cancer Ther.

[12] Peters AA, Buchanan G, Ricciardelli C, Bianco-Miotto T, Centenera MM, Harris JM, Jindal S, Segara D, Jia L, Moore NL (2009). Androgen receptor inhibits estrogen receptor-alpha activity and is prognostic in breast cancer. Cancer Res.

[13] Robinson JL, Macarthur S, Ross-Innes CS, Tilley WD, Neal DE, Mills IG, Carroll JS (2011). Androgen receptor driven transcription in molecular apocrine breast cancer is mediated by FoxA1. EMBO J.

[14] Gerratana L, Basile D, Buono G, De Placido S, Giuliano M, Minichillo S, Coinu A, Martorana F, De Santo I, Del Mastro L (2018). Androgen receptor in triple negative breast cancer: a potential target for the targetless subtype. Cancer Treat Rev.

[15] Morozzi C, Sedlakova J, Serpi M, Avigliano M, Carbajo R, Sandoval L, Valles-Ayoub Y, Crutcher P, Thomas S, Pertusati F (2019). Targeting GNE myopathy: a dual prodrug approach for the delivery of N-Acetylmannosamine 6-phosphate. J Med Chem.

[16] Barton VN, D'Amato NC, Gordon MA, Christenson JL, Elias A, Richer JK (2015). Androgen receptor biology in triple negative breast Cancer: a case for classification as AR+ or quadruple negative disease. Horm Cancer.

[17] Gristina V, La Mantia M, Iacono F, Galvano A, Russo A, Bazan V. The emerging therapeutic landscape of ALK inhibitors in non-small cell lung Cancer. Pharmaceuticals (Basel). 2020;13(12). 10.3390/ph13120474 PMC7766858 33352844

[18] Doane AS, Danso M, Lal P, Donaton M, Zhang L, Hudis C, Gerald WL (2006). An estrogen receptor-negative breast cancer subset characterized by a hormonally regulated transcriptional program and response to androgen. Oncogene.

[19] Kikuchi K, McNamara KM, Miki Y, Moon JY, Choi MH, Omata F, Sakurai M, Onodera Y, Rai Y, Ohi Y (2017). Effects of cytokines derived from cancer-associated fibroblasts on androgen synthetic enzymes in estrogen receptor-negative breast carcinoma. Breast Cancer Res Treat.

[20] Verduzco D, Kuenzi BM, Kinose F, Sondak VK, Eroglu Z, Rix U, Smalley KSM (2018). Ceritinib enhances the efficacy of Trametinib in BRAF/NRAS-wild-type melanoma cell lines. Mol Cancer Ther.

[21] Kuenzi BM, Remsing Rix LL, Stewart PA, Fang B, Kinose F, Bryant AT, Boyle TA, Koomen JM, Haura EB, Rix U (2017). Polypharmacology-based ceritinib repurposing using integrated functional proteomics. Nat Chem Biol.

[22] Shaw AT, Kim DW, Mehra R, Tan DS, Felip E, Chow LQ, Camidge DR, Vansteenkiste J, Sharma S, De Pas T (2014). Ceritinib in ALK-rearranged non-small-cell lung cancer. N Engl J Med.

[23] Ekyalongo RC, Yee D. Revisiting the IGF-1R as a breast cancer target. NPJ precis Oncol. 2017;1. 10.1038/s41698-017-0017-y PMC5687252 29152592

[24] Wu X, Zahari MS, Renuse S, Kelkar DS, Barbhuiya MA, Rojas PL, Stearns V, Gabrielson E, Malla P, Sukumar S (2017). The non-receptor tyrosine kinase TNK2/ACK1 is a novel therapeutic target in triple negative breast cancer. Oncotarget.

[25] Luo M, Guan JL (2010). Focal adhesion kinase: a prominent determinant in breast cancer initiation, progression and metastasis. Cancer Lett.

[26] Al-Aidaroos AQ, Yuen HF, Guo K, Zhang SD, Chung TH, Chng WJ, Zeng Q (2013). Metastasis-associated PRL-3 induces EGFR activation and addiction in cancer cells. J Clin Invest.

[27] Wu X, Zahari MS, Ma B, Liu R, Renuse S, Sahasrabuddhe NA, Chen L, Chaerkady R, Kim MS, Zhong J (2015). Global phosphotyrosine survey in triple-negative breast cancer reveals activation of multiple tyrosine kinase signaling pathways. Oncotarget.

[28] Mahajan NP, Liu Y, Majumder S, Warren MR, Parker CE, Mohler JL, Earp HS, Whang YE (2007). Activated Cdc42-associated kinase Ack1 promotes prostate cancer progression via androgen receptor tyrosine phosphorylation. Proc Natl Acad Sci U S A.

[29] Butti R, Das S, Gunasekaran VP, Yadav AS, Kumar D, Kundu GC (2018). Receptor tyrosine kinases (RTKs) in breast cancer: signaling, therapeutic implications and challenges. Mol Cancer.

[30] Mahajan K, Malla P, Lawrence HR, Chen Z, Kumar-Sinha C, Malik R, Shukla S, Kim J, Coppola D, Lawrence NJ (2017). ACK1/TNK2 regulates histone H4 Tyr88-phosphorylation and AR gene expression in castration-resistant prostate Cancer. Cancer Cell.

[31] Yang L, Li Y, Shen E, Cao F, Li L, Li X, Wang X, Kariminia S, Chang B, Li H (2017). NRG1-dependent activation of HER3 induces primary resistance to trastuzumab in HER2-overexpressing breast cancer cells. Int J Oncol.

[32] Sak MM, Breen K, Ronning SB, Pedersen NM, Bertelsen V, Stang E, Madshus IH (2012). The oncoprotein ErbB3 is endocytosed in the absence of added ligand in a clathrin-dependent manner. Carcinogenesis.

[33] Ni M, Chen Y, Lim E, Wimberly H, Bailey ST, Imai Y, Rimm DL, Liu XS, Brown M (2011). Targeting androgen receptor in estrogen receptor-negative breast cancer. Cancer Cell.

[34] Barton VN, Christenson JL, Gordon MA, Greene LI, Rogers TJ, Butterfield K, Babbs B, Spoelstra NS, D'Amato NC, Elias A (2017). Androgen receptor supports an Anchorage-independent, Cancer stem cell-like population in triple-negative breast Cancer. Cancer Res.

[35] Baranwal S, Wang Y, Rathinam R, Lee J, Jin L, McGoey R, Pylayeva Y, Giancotti F, Blobe GC, Alahari SK (2011). Molecular characterization of the tumor-suppressive function of nischarin in breast cancer. J Natl Cancer Inst.

[36] Liu Z, Adams HC, Whitehead IP (2009). The rho-specific guanine nucleotide exchange factor Dbs regulates breast cancer cell migration. J Biol Chem.

[37] Narayan P, Wahby S, Gao JJ, Amiri-Kordestani L, Ibrahim A, Bloomquist E, Tang S, Xu Y, Liu J, Fu W (2020). FDA approval summary: Atezolizumab plus paclitaxel protein-bound for the treatment of patients with advanced or metastatic TNBC whose tumors express PD-L1. Clin Cancer Res.

[38] Jurj A, Pop LA, Zanoaga O, Ciocan-Cartita CA, Cojocneanu R, Moldovan C, Raduly L, Pop-Bica C, Trif M, Irimie A (2020). New insights in gene expression alteration as effect of paclitaxel drug resistance in triple negative breast Cancer cells. Cell Physiol Biochem.

[39] Kang Y, Hu W, Ivan C, Dalton HJ, Miyake T, Pecot CV, Zand B, Liu T, Huang J, Jennings NB (2013). Role of focal adhesion kinase in regulating YB-1-mediated paclitaxel resistance in ovarian cancer. J Natl Cancer Inst.

[40] Tancioni I, Miller NL, Uryu S, Lawson C, Jean C, Chen XL, Kleinschmidt EG, Schlaepfer DD (2015). FAK activity protects nucleostemin in facilitating breast cancer spheroid and tumor growth. Breast Cancer Res.

[41] Pop L, Suciu I, Ionescu O, Bacalbasa N, Ionescu P (2021). The role of novel poly (ADP-ribose) inhibitors in the treatment of locally advanced and metastatic Her-2/neu negative breast cancer with inherited germline BRCA1/2 mutations. A review of the literature. J Med Life.

[42] Steiner M, Tan AR (2021). The evolving role of immune checkpoint inhibitors in the treatment of triple-negative breast cancer. Clin Adv Hematol Oncol.

[43] Bartsch R, ESMO (2020). Highlights in breast cancer. Memo.

[44] Nagayama A, Vidula N, Bardia A (2021). Novel therapies for metastatic triple-negative breast Cancer: spotlight on immunotherapy and antibody-drug conjugates. Oncology (Williston Park).

[45] Won KA, Spruck C (2020). Triplenegative breast cancer therapy: current and future perspectives (review). Int J Oncol.

[46] Ding YC, Steele L, Warden C, Wilczynski S, Mortimer J, Yuan Y, Neuhausen SL (2019). Molecular subtypes of triple-negative breast cancer in women of different race and ethnicity. Oncotarget.

[47] Davey RA, Grossmann M (2016). Androgen receptor structure, function and biology: from bench to bedside. Clin Biochem Rev.

[48] Labrie F, Luu-The V, Labrie C, Belanger A, Simard J, Lin SX, Pelletier G (2003). Endocrine and intracrine sources of androgens in women: inhibition of breast cancer and other roles of androgens and their precursor dehydroepiandrosterone. Endocr Rev.

[49] Vermeulen A (1998). Plasma androgens in women. J Reprod Med.

[50] Davison SL, Bell R, Donath S, Montalto JG, Davis SR (2005). Androgen levels in adult females: changes with age, menopause, and oophorectomy. J Clin Endocrinol Metab.

[51] Lehmann BD, Bauer JA, Schafer JM, Pendleton CS, Tang L, Johnson KC, Chen X, Balko JM, Gomez H, Arteaga CL (2014). PIK3CA mutations in androgen receptor-positive triple negative breast cancer confer sensitivity to the combination of PI3K and androgen receptor inhibitors. Breast Cancer Res.

[52] Tan MH, Li J, Xu HE, Melcher K, Yong EL (2015). Androgen receptor: structure, role in prostate cancer and drug discovery. Acta Pharmacol Sin.

[53] Friboulet L, Li N, Katayama R, Lee CC, Gainor JF, Crystal AS, Michellys PY, Awad MM, Yanagitani N, Kim S (2014). The ALK inhibitor ceritinib overcomes crizotinib resistance in non-small cell lung cancer. Cancer Discov.

