# Supplementary material for: Ceritinib is a novel triple negative breast cancer therapeutic agent
Source: Mol Cancer. 2022 Jun 29;21:138. doi: 10.1186/s12943-022-01601-0 (PMC9241294; doi:10.1186/s12943-022-01601-0)
Supplement: Supplementary file 1 [file 12943_2022_1601_MOESM10_ESM.docx]

SFig. 10

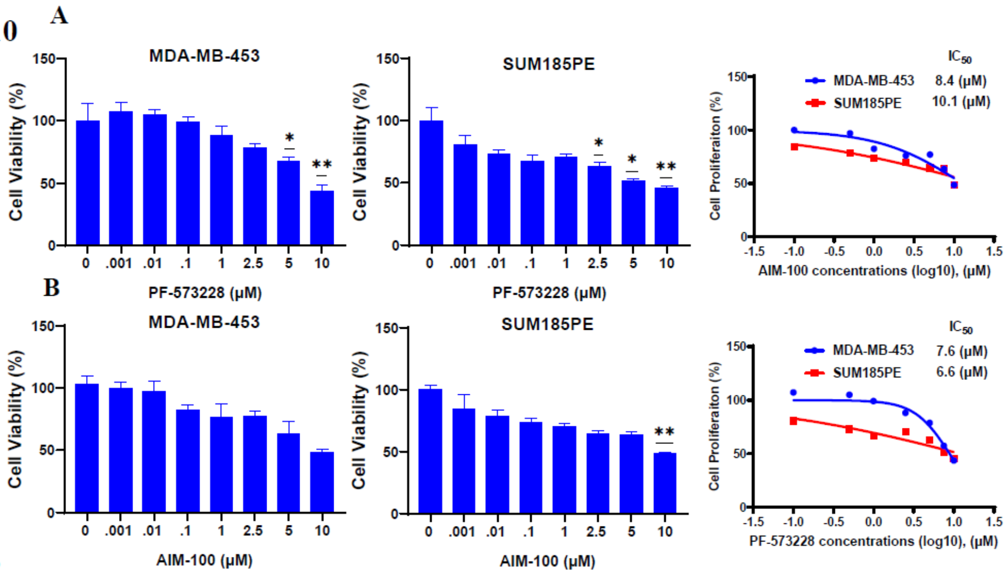

Figure S10

PF-573228 and AIM-100 inhibited TNBC cell growth. (A) The effects of the FAK inhibitor (PF-573228) on MDA-MB-453 and SUM185PE proliferation were estimated by MTT assay after 24 h of treatment. IC50 values were collected from three independent experiments, each performed in triplicate. IC50 is the concentration of drug that caused a 50% reduction in proliferation compared to the vehicle-treated cells. **(B)** The effects of the ACK1 inhibitor (AIM-100) on MDA-MB-453 and SUM185PE proliferation were estimated by MTT assay after 24 h of treatment.
