# Supplementary material for: Ceritinib is a novel triple negative breast cancer therapeutic agent
Source: Mol Cancer. 2022 Jun 29;21:138. doi: 10.1186/s12943-022-01601-0 (PMC9241294; doi:10.1186/s12943-022-01601-0)
Supplement: Supplementary file 2 [file 12943_2022_1601_MOESM11_ESM.docx]

SFig. 11

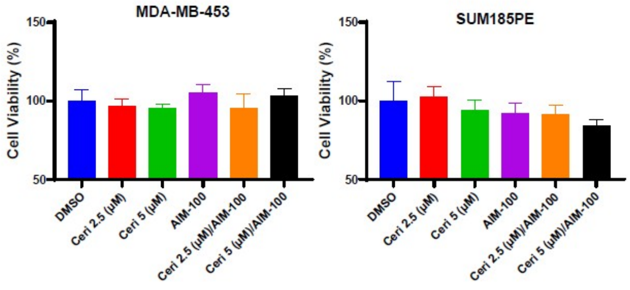
MDA-MB-453
SUM185PE
Cell Viability (%)
150
100
50
DMSO
Ceri 2.5 (μM)
Ceri 5 (μM)
AIM-100
Ceri 2.5 (μM)/AIM-100
Ceri 5 (μM)/AIM-100
