# Supplementary material for: Ceritinib is a novel triple negative breast cancer therapeutic agent
Source: Mol Cancer. 2022 Jun 29;21:138. doi: 10.1186/s12943-022-01601-0 (PMC9241294; doi:10.1186/s12943-022-01601-0)
Supplement: Supplementary file 3 [file 12943_2022_1601_MOESM12_ESM.docx]

SFig. 12

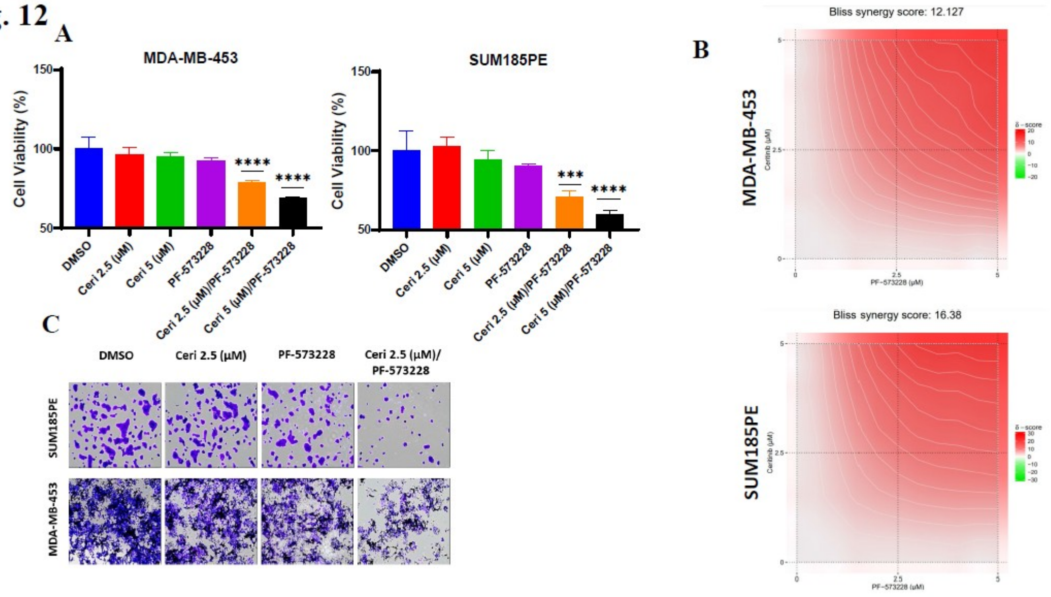
A
MDA-MB-453
SUM185PE
Cell Viability (%)
150
100
50
DMSO
Ceri 2.5 (µM)
Ceri 5 (µM)
PF-573228
Ceri 2.5 (µM)/PF-573228
Ceri 5 (µM)/PF-573228
****
***
B
Bliss synergy score: 12.127
Bliss synergy score: 16.38
Ceritinib (µM)
PF−573228 (µM)
δ - score
C
DMSO
Ceri 2.5 (µM)
PF-573228
Ceri 2.5 (µM)/ PF-573228
SUM185PE
MDA-MB-453
