# Supplementary material for: Ceritinib is a novel triple negative breast cancer therapeutic agent
Source: Mol Cancer. 2022 Jun 29;21:138. doi: 10.1186/s12943-022-01601-0 (PMC9241294; doi:10.1186/s12943-022-01601-0)
Supplement: Supplementary file 4 [file 12943_2022_1601_MOESM13_ESM.docx]

SFig. 13

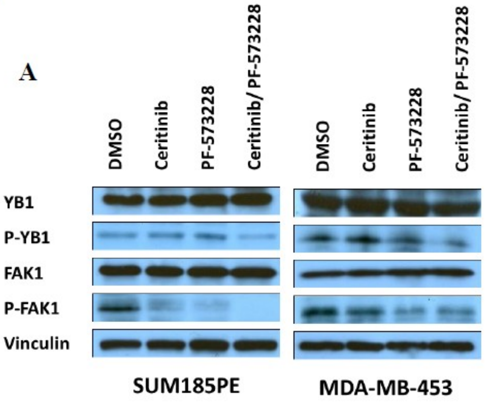

A
DMSO
Ceritinib
PF-573228
Ceritinib/ PF-573228
DMSO
Ceritinib
PF-573228
Ceritinib/ PF-573228
YB1
P-YB1
FAK1
P-FAK1
Vinculin
SUM185PE
MDA-MB-453

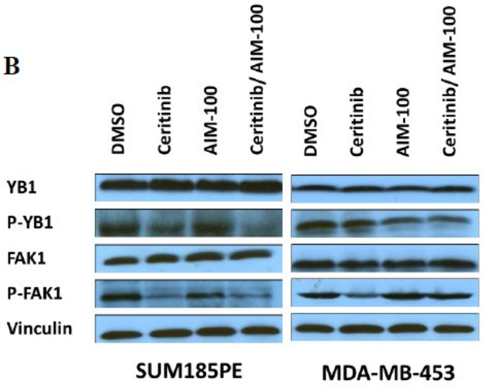

B
DMSO
Ceritinib
AIM-100
Ceritinib/ AIM-100
DMSO
Ceritinib
AIM-100
Ceritinib/ AIM-100
YB1
P-YB1
FAK1
P-FAK1
Vinculin
SUM185PE
MDA-MB-453
