# Supplementary material for: Ceritinib is a novel triple negative breast cancer therapeutic agent
Source: Mol Cancer. 2022 Jun 29;21:138. doi: 10.1186/s12943-022-01601-0 (PMC9241294; doi:10.1186/s12943-022-01601-0)
Supplement: Supplementary file 5 [file 12943_2022_1601_MOESM14_ESM.docx]

SFig. 14

A

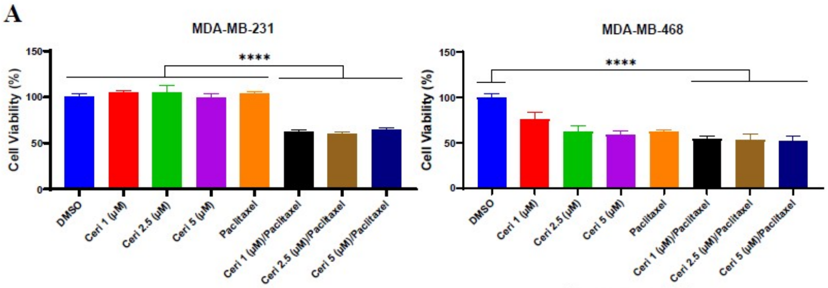

MDA-MB-231
MDA-MB-468
Cell Viability (%)
****
DMSO
Ceri 1 (μM)
Ceri 2.5 (μM)
Ceri 5 (μM)
Paclitaxel
Ceri 1 (μM)/Paclitaxel
Ceri 2.5 (μM)/Paclitaxel
Ceri 5 (μM)/Paclitaxel

B

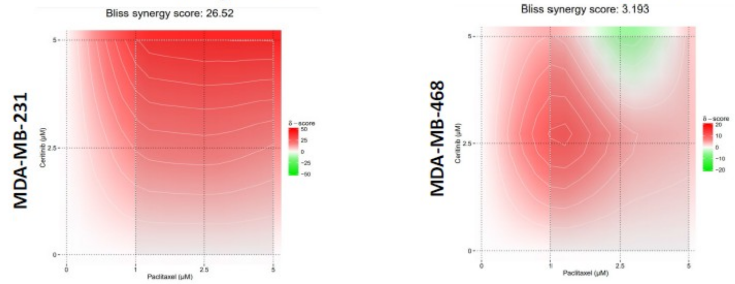

Bliss synergy score: 26.52
MDA-MB-231
Bliss synergy score: 3.193
MDA-MB-468
Ceritinib (μM)
Paclitaxel (μM)
δ - score
