# Supplementary material for: Ceritinib is a novel triple negative breast cancer therapeutic agent
Source: Mol Cancer. 2022 Jun 29;21:138. doi: 10.1186/s12943-022-01601-0 (PMC9241294; doi:10.1186/s12943-022-01601-0)
Supplement: Supplementary file 6 [file 12943_2022_1601_MOESM15_ESM.docx]

SFig. 15

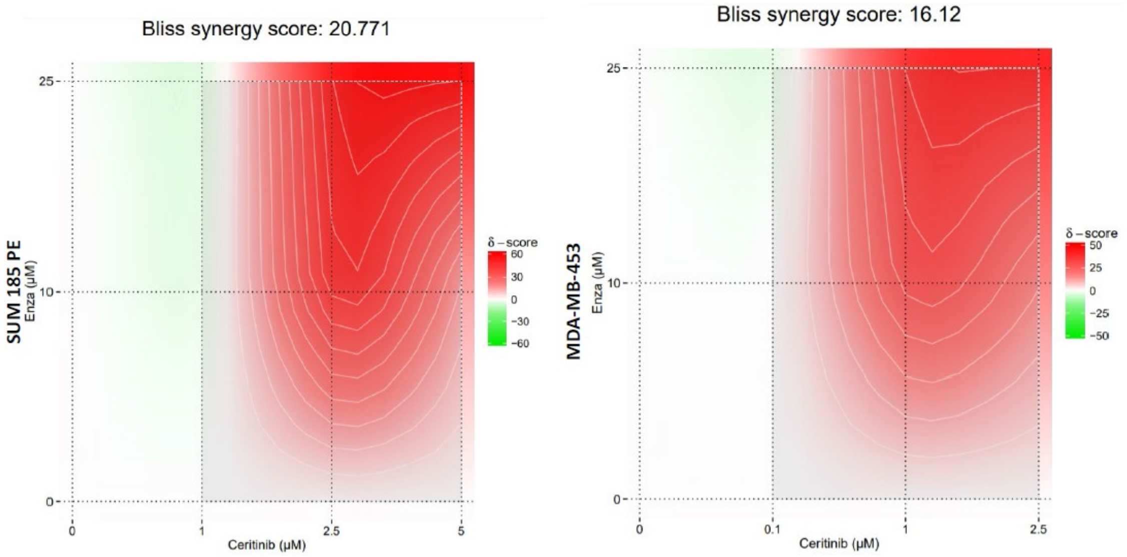
Bliss synergy score: 20.771
SUM 185 PE
Enza (μM)
25
10
0
Ceritinib (μM)
0
1
2.5
5
δ – score
60
30
0
−30
−60
Bliss synergy score: 16.12
MDA-MB-453
Enza (μM)
25
10
0
Ceritinib (μM)
0
0.1
1
2.5
δ – score
50
25
0
−25
−50
