# Supplementary material for: Ceritinib is a novel triple negative breast cancer therapeutic agent
Source: Mol Cancer. 2022 Jun 29;21:138. doi: 10.1186/s12943-022-01601-0 (PMC9241294; doi:10.1186/s12943-022-01601-0)
Supplement: Supplementary file 7 [file 12943_2022_1601_MOESM1_ESM.docx]

S Fig.1

A

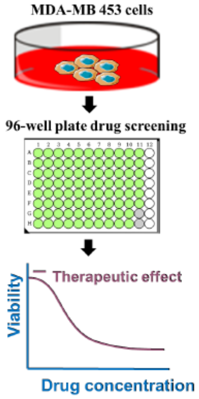

B

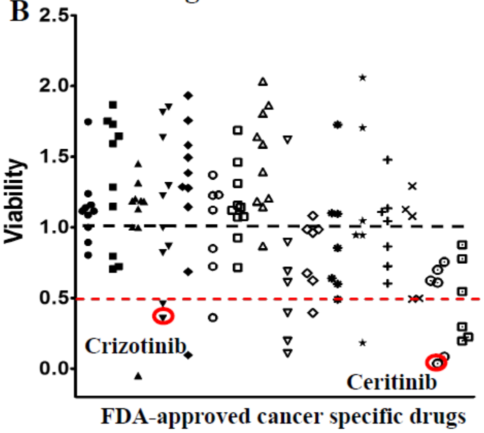

C

Cell Viability	<10%	<20%	<30%	<40%	<50%
Drugs	Mitomycin	Cyclophosphamide	Daunorubicin	Sorafenid	Crizotinib
	Mitoxantrone	Romidepsin	Valrubicin	Afatinib	Bosutinib
	Idarubicin	Doxorubicin		Vinorelbine	Lapatinib
	Ceritinib	Dactinomycin		Vinblastine	Ixazomib
