# Supplementary material for: Ceritinib is a novel triple negative breast cancer therapeutic agent
Source: Mol Cancer. 2022 Jun 29;21:138. doi: 10.1186/s12943-022-01601-0 (PMC9241294; doi:10.1186/s12943-022-01601-0)
Supplement: Supplementary file 8 [file 12943_2022_1601_MOESM2_ESM.docx]

SFig. 2

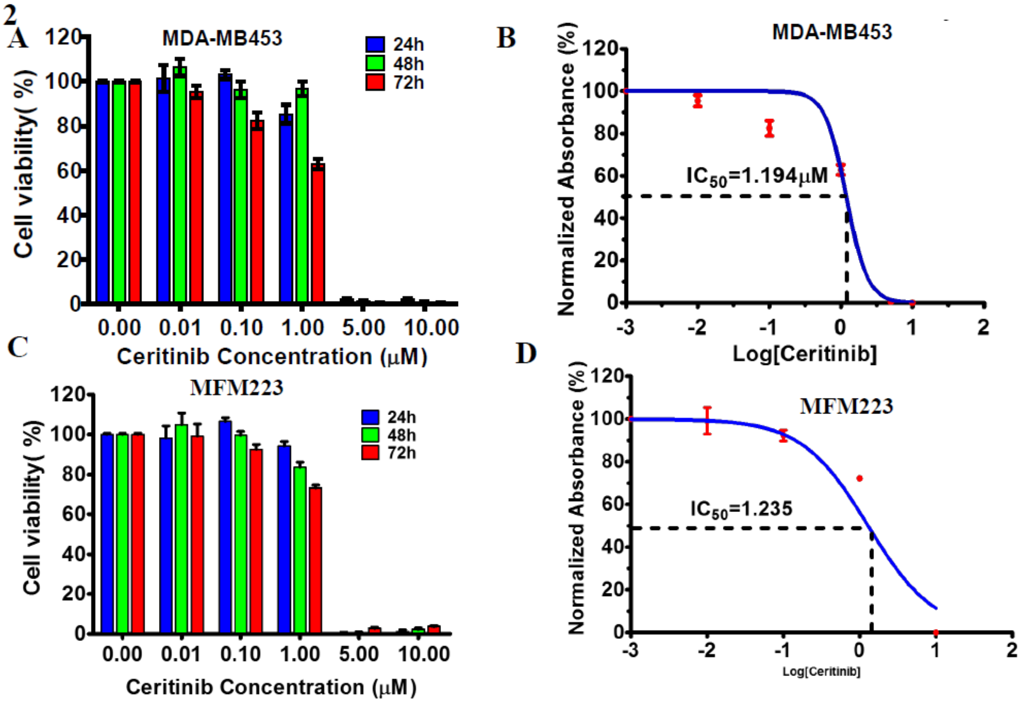
A
MDA-MB453
24h
48h
72h
Cell viability(%)
120
100
80
60
40
20
0
0.00
0.01
0.10
1.00
5.00
10.00
Ceritinib Concentration (μM)
B
MDA-MB453
Normalized Absorbance (%)
IC50=1.194μM
Log[Ceritinib]
C
MFM223
Ceritinib Concentration (μM)
D
MFM223
IC50=1.235
Log[Ceritinib]
