# Supplementary material for: Ceritinib is a novel triple negative breast cancer therapeutic agent
Source: Mol Cancer. 2022 Jun 29;21:138. doi: 10.1186/s12943-022-01601-0 (PMC9241294; doi:10.1186/s12943-022-01601-0)
Supplement: Supplementary file 9 [file 12943_2022_1601_MOESM3_ESM.docx]

A

SFig. 3

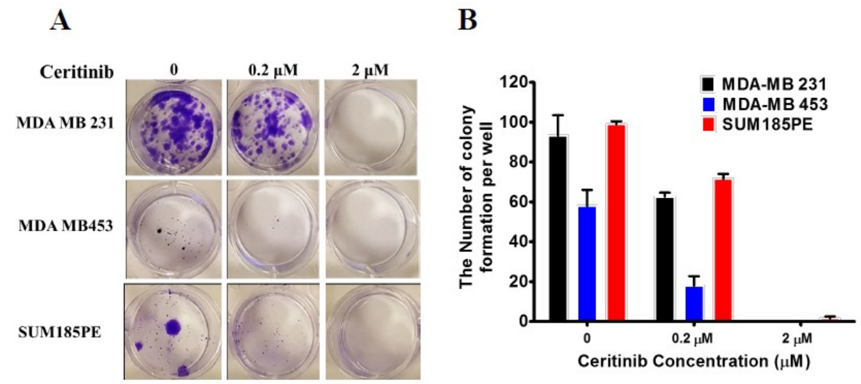
A
Ceritinib
0
0.2 μM
2 μM
MDA MB 231
MDA MB453
SUM185PE
B
The Number of colony formation per well
120
100
80
60
40
20
0
MDA-MB 231
MDA-MB 453
SUM185PE
0
0.2 μM
2 μM
Ceritinib Concentration (μM)
