# Supplementary material for: Ceritinib is a novel triple negative breast cancer therapeutic agent
Source: Mol Cancer. 2022 Jun 29;21:138. doi: 10.1186/s12943-022-01601-0 (PMC9241294; doi:10.1186/s12943-022-01601-0)
Supplement: Supplementary file 10 [file 12943_2022_1601_MOESM4_ESM.docx]

SFig. 4

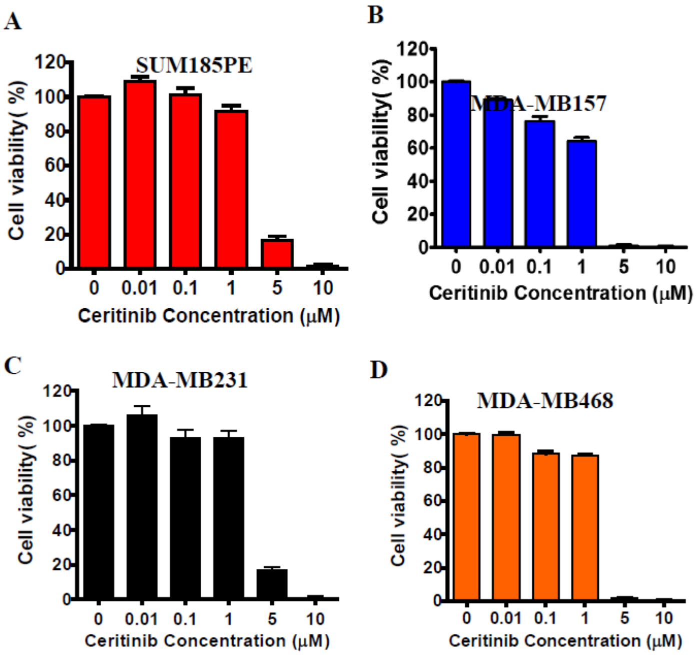
A
SUM185PE
Cell viability(%)
120
100
80
60
40
20
0
0
0.01
0.1
1
5
10
Ceritinib Concentration (μM)
B
MDA-MB157
Cell viability(%)
Ceritinib Concentration (μM)
C
MDA-MB231
Cell viability(%)
Ceritinib Concentration (μM)
D
MDA-MB468
Cell viability(%)
Ceritinib Concentration (μM)
