# Supplementary material for: Ceritinib is a novel triple negative breast cancer therapeutic agent
Source: Mol Cancer. 2022 Jun 29;21:138. doi: 10.1186/s12943-022-01601-0 (PMC9241294; doi:10.1186/s12943-022-01601-0)
Supplement: Supplementary file 12 [file 12943_2022_1601_MOESM6_ESM.docx]

SFig. 6

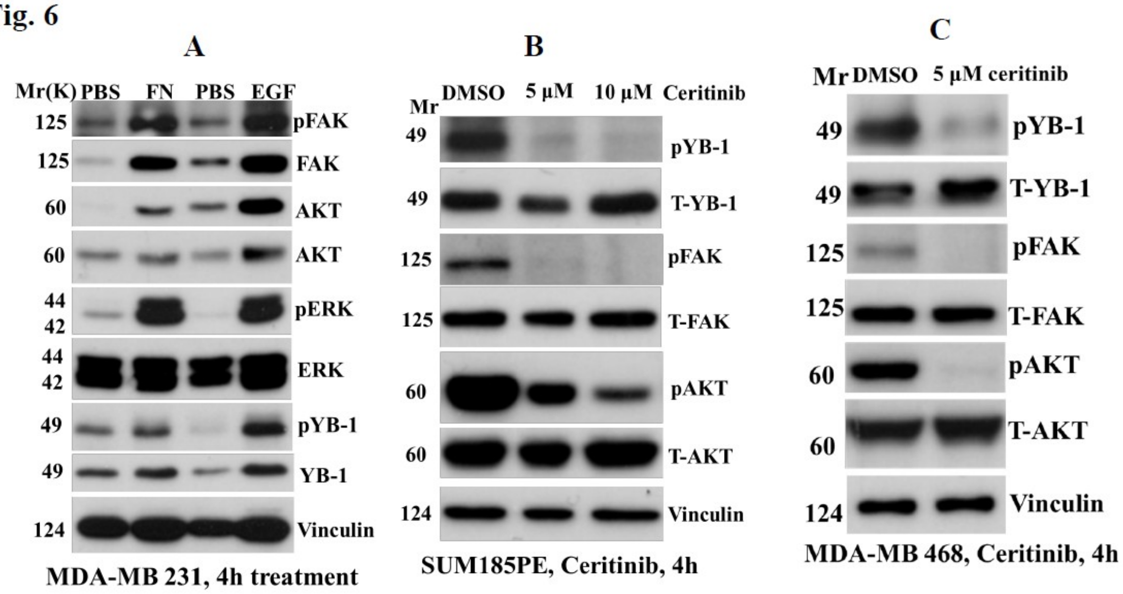
A
Mr(K) PBS FN PBS EGF
125 pFAK
125 FAK
60 AKT
60 AKT
44 42 pERK
44 42 ERK
49 pYB-1
49 YB-1
124 Vinculin
MDA-MB 231, 4h treatment
B
Mr DMSO 5 µM 10 µM Ceritinib
49 pYB-1
49 T-YB-1
125 pFAK
125 T-FAK
60 pAKT
60 T-AKT
124 Vinculin
SUM185PE, Ceritinib, 4h
C
Mr DMSO 5 µM ceritinib
49 pYB-1
49 T-YB-1
125 pFAK
125 T-FAK
60 pAKT
60 T-AKT
124 Vinculin
MDA-MB 468, Ceritinib, 4h
