# Supplementary material for: Ceritinib is a novel triple negative breast cancer therapeutic agent
Source: Mol Cancer. 2022 Jun 29;21:138. doi: 10.1186/s12943-022-01601-0 (PMC9241294; doi:10.1186/s12943-022-01601-0)
Supplement: Supplementary file 13 [file 12943_2022_1601_MOESM7_ESM.docx]

SFig. 7

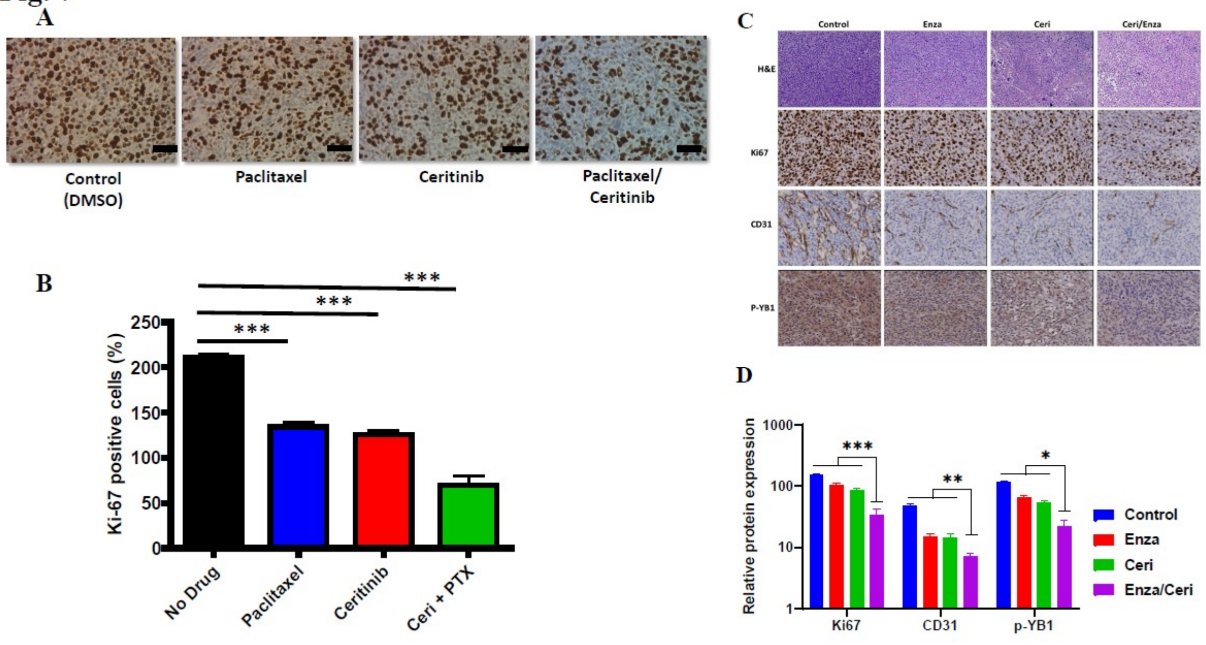

A
Control (DMSO)
Paclitaxel
Ceritinib
Paclitaxel/ Ceritinib
B
Ki-67 positive cells (%)
250
200
150
100
50
0
***
No Drug
Paclitaxel
Ceritinib
Ceri + PTX
C
Control
Enza
Ceri
Ceri/Enza
H&E
Ki67
CD31
P-YB1
D
Relative protein expression
1000
100
10
1
***
**
*
Ki67
CD31
p-YB1
Control
Enza
Ceri
Enza/Ceri
