# Supplementary material for: Ceritinib is a novel triple negative breast cancer therapeutic agent
Source: Mol Cancer. 2022 Jun 29;21:138. doi: 10.1186/s12943-022-01601-0 (PMC9241294; doi:10.1186/s12943-022-01601-0)

SFig. 8

A

PDX Model	PAM50 Subtype	Histologic Type	Age	Ethnicity	Tumor Grade	LN Involvement
Tu-2K1	TNBC	Invasive ductal	59	AA	---	N
Tu-4EALNb	TNBC	Invasive ductal	58	AA	3	Y (3/13 nodes)
Tu-4IC	TNBC	Metaplastic carcinoma	57	Cauc	3	Y (1/17 nodes)
Tu-4M4Tb	TNBC	Invasive carcinoma	63	AA	3	Y (multiple)
Tu-4QXTb	TNBC	Invasive carcinoma	N/A	AA	2	Y (11/15 nodes)

B

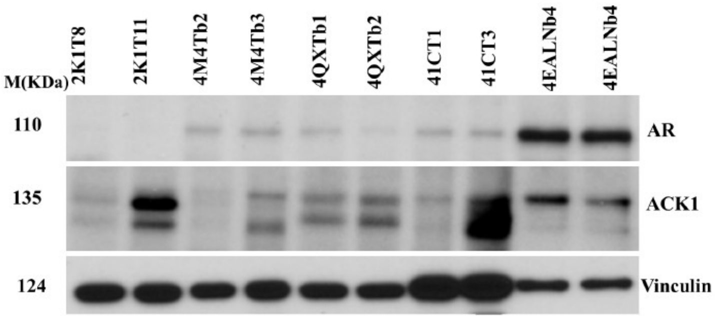

Supplement: Supplementary file 14 [file 12943_2022_1601_MOESM8_ESM.docx]
