# Supplementary material for: Ceritinib is a novel triple negative breast cancer therapeutic agent
Source: Mol Cancer. 2022 Jun 29;21:138. doi: 10.1186/s12943-022-01601-0 (PMC9241294; doi:10.1186/s12943-022-01601-0)
Supplement: Supplementary file 15 [file 12943_2022_1601_MOESM9_ESM.docx]

SFig. 9

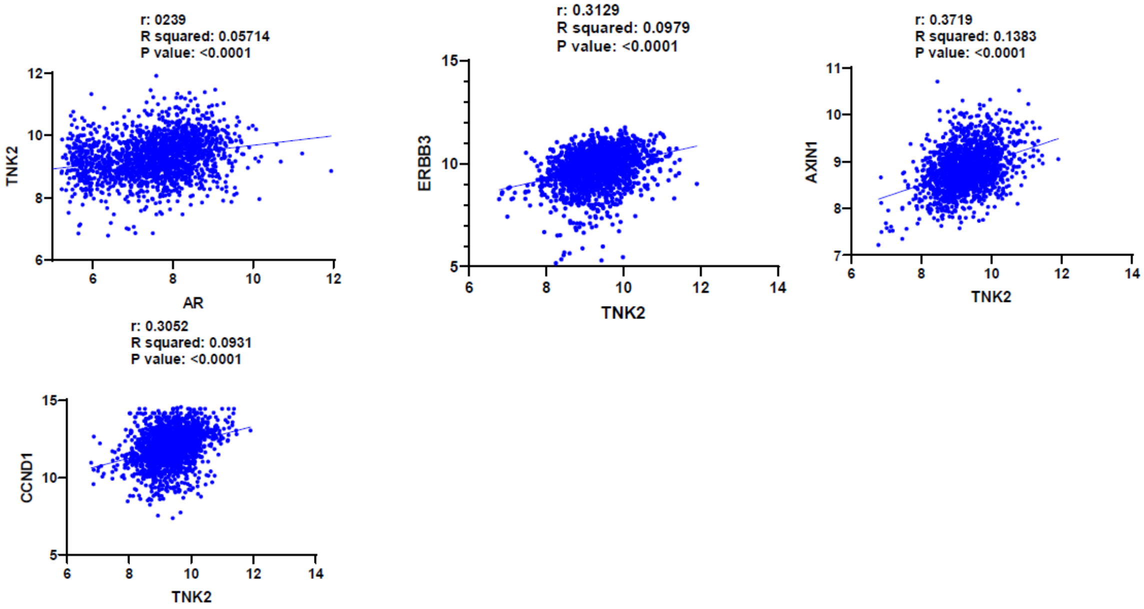
r: 0239
R squared: 0.05714
P value: <0.0001
TNK2
AR
r: 0.3129
R squared: 0.0979
P value: <0.0001
ERBB3
TNK2
r: 0.3719
R squared: 0.1383
P value: <0.0001
AXIN1
TNK2
r: 0.3052
R squared: 0.0931
P value: <0.0001
CCND1
TNK2
